# Urolithin A provides cardioprotection and mitochondrial quality enhancement preclinically and improves human cardiovascular health biomarkers

**DOI:** 10.1016/j.isci.2025.111814

**Published:** 2025-01-14

**Authors:** Sophia Liu, Julie Faitg, Charlotte Tissot, Dimitris Konstantopoulos, Ross Laws, Guillaume Bourdier, Pénélope A. Andreux, Tracey Davey, Hector Gallart-Ayala, Julijana Ivanisevic, Anurag Singh, Chris Rinsch, David J. Marcinek, Davide D’Amico

**Affiliations:** 1 Department of Radiology, University of Washington Medical Center, Box 358050, Seattle, WA 98109, USA; 2 Amazentis, EPFL Innovation Park, Lausanne, Switzerland; 3 Genevia Technologies Oy, 33100 Tampere, Finland; 4 Electron Microscopy Research Services, Newcastle University, Newcastle, UK; 5 Syncrosome, Campus Luminy - Luminy Entreprises, 13288 Marseille, France; 6 Vandria, EPFL Innovation Park, Lausanne, Switzerland; 7 Metabolomics Platform, Faculty of Biology and Medicine, University of Lausanne, Lausanne, Switzerland

**Keywords:** Biological sciences, Cardiovascular medicine, Health sciences, Internal medicine, Medical specialty, Medicine, Natural sciences, Physiology

## Abstract

Cardiovascular diseases (CVDs) remain the primary cause of global mortality. Nutritional interventions hold promise to reduce CVD risks in an increasingly aging population. However, few nutritional interventions are proven to support heart health and act mostly on blood lipid homeostasis rather than at cardiac cell level. Here, we show that mitochondrial quality dysfunctions are common hallmarks in human cardiomyocytes upon heart aging and in chronic conditions. Preclinically, the post-biotic and mitophagy activator, urolithin A (UA), reduced both systolic and diastolic cardiac dysfunction in models of natural aging and heart failure. At a cellular level, this was associated with a recovery of mitochondrial ultrastructural defects and mitophagy. In humans, UA supplementation for 4 months in healthy older adults significantly reduced plasma ceramides clinically validated to predict CVD risks. These findings extend and translate UA’s benefits to heart health, making UA a promising nutritional intervention to support cardiovascular function as we age.

## Highlights

• Urolithin A enhances heart mitochondrial quality in aging and heart failure models
• Cardiac function decline in these models is reduced by urolithin A
• In humans, urolithin A lowers plasma ceramides associated with heart disease risk
• Urolithin A is a promising nutritional approach to support heart health as we age

## Graphical abstract

**Figure:**
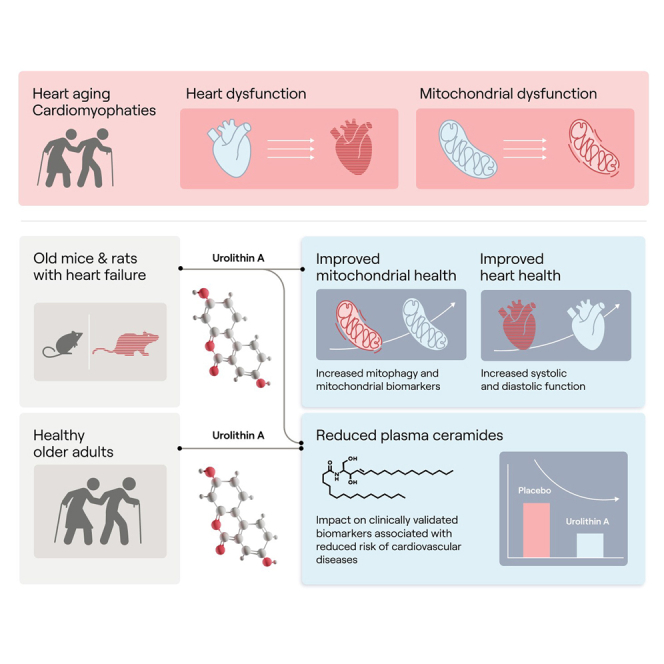

## Introduction

Cardiovascular disease (CVD) results in over $320 billion in public health spending per year^1^ and age is one of the greatest risk factors.^2^ Age-related changes of the heart include thickening and stiffening of the left ventricular walls, increased fibrosis, and metabolic disruptions. These changes leads to decline in cardiac function,^3^^,^^4^ exercise intolerance and ultimately a poorer quality of life.^5^^,^^6^^,^^7^ While drugs and strategies exist for managing heart disease, there remains a critical need to develop preventive interventions to reduce cardiac morbidities as we age.

Nutritional supplements specifically targeting heart health, in conjunction with a healthy lifestyle, offer a promising approach to reducing CVD risks. However, most current supplements have limited evidence of efficacy in humans and typically target heart health indirectly by reducing lipid and cholesterol levels. There is a lack of interventions that directly address cardiomyocyte dysfunctions and can be safely translated into heart health benefits for humans.

Given the high ATP demand of the heart, 90% of which goes to support Ca^2+^ pumping and cross-bridge cycling, the heart is especially sensitive to impaired mitochondrial energetics.^8^ Preclinical studies have shown that mitochondrial dysfunction is a key driver of functional decline in the aging heart by negatively impacting cardiomyocyte bioenergetics.^9^^,^^10^^,^^11^ Aging is associated with the accumulation of poorly functioning mitochondria, which is linked to a decline in mitochondrial recycling by mitophagy.^12^^,^^13^^,^^14^

In our study, we first confirmed that mitochondrial and mitophagy dysfunctions are common hallmarks of human cardiac aging and disease. We then investigated the potential benefits for heart health of the mitophagy booster urolithin A (UA), a naturally derived postbiotic produced by the gut microbiome from ellagitannins found in many fruits and nuts.^15^ UA has already been observed to activate mitophagy, and improve healthspan in several models of aging and disease.^16^^,^^17^^,^^18^ Moreover, UA supplementation for cardiac function has promising translational potential, with three randomized clinical trials already showing that UA is safe and improves skeletal muscle function in older adults,^19^ and in overweight, middle-aged adults.^20^ Our research expanded on these findings by testing UA’s impact on cardiac dysfunction and mitochondrial health in two preclinical models: a mouse model of natural aging partially typified by preserved ejection fraction (EF), and a rat heart failure model, characterized by reduced ejection fraction (HFrEF). Finally, we evaluated the potential of UA for human heart health by studying its beneficial impact on ceramide blood biomarkers associated with increased CVD risk in a randomized clinical study involving healthy older subjects.

## Results

### Mitochondrial dysfunction is a common feature of heart aging and disease in humans

**Figure 1:**
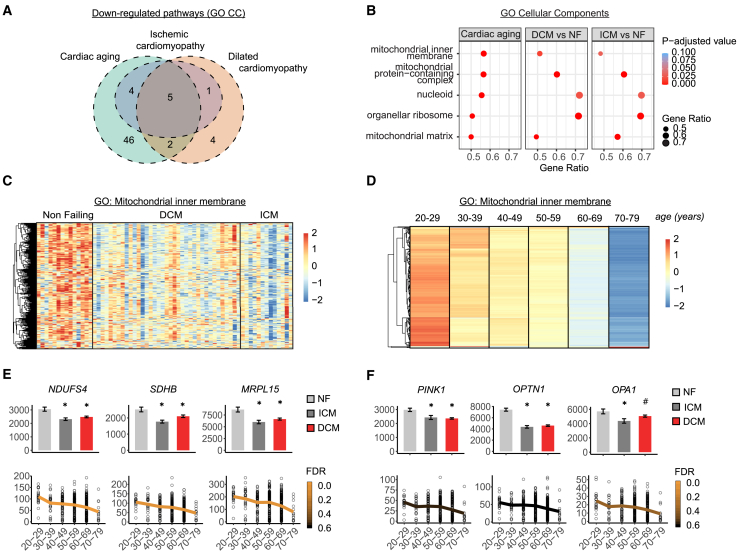
Mitochondrial dysfunction is a common hallmark of human cardiac aging and disease (A) Venn diagram depicting overlap of significantly downregulated pathways (Gene Ontology cellular components [GO CCs]) among three human datasets: cardiac aging, dilated cardiomyopathy (DCM), and ischemic cardiomyopathy (ICM). GO CCs were identified using gene set enrichment analysis (GSEA) and categorized as “downregulated” based on their normalized enrichment scores. (B) Dot plot of five commonly suppressed GO CCs shared among all three datasets in (A). GO CCs are sorted by “cardiac aging” gene ratio. The size of each dot corresponds to the gene ratio, while the color indicates the statistical significance of the enrichment (Benjamini-Hochberg-adj. *p* value). (C) Heatmaps of scaled normalized expression counts (*Z* scores) of genes that contributed to the enrichment of the “mitochondrial inner membrane” GO CC term in human DCM and ICM compared to non-failing. Rows represent genes hierarchically clustered using a Euclidean distance metric and complete linkage. Columns represent individual samples from each condition. (D) Heatmaps of the scaled transcripts per million (TPM, as *Z* scores) of core enrichment genes contributing to the “mitochondrial inner membrane” GO CC term in the human cardiac aging study (GTEx aging). Rows represent genes hierarchically clustered using a Euclidean distance metric and complete linkage. Columns represent different age groups, sorted in ascending order. For each age group, mean TPM values were calculated for all genes before scaling. (E) Expression levels of top three most significantly downregulated genes in the “mitochondrial inner membrane” category comparing cardiac diseases (ICM and DCM) to controls (NF) (bar plot, top) or comparing age groups (scatterplot, bottom). For top panels adjusted *p* value is corrected for multiple-testing using the Benjamini and Hochberg (BH) method. ∗*p* < 0.05. Exact *p* values: *NDUFS4* ICM vs. NF, *p* = 0.0001, *NDUFS4* DCM vs. NF, *p* = 0.027. *SDHB* ICM vss. NF, *p* = 0.00009, *SDHB* DCM vs. NF, *p* = 0.0005. *MRPL15* ICM vss. NF, *p* = 0.0001, *MRPL15* DCM vs. NF, *p* = 0.0004. Lower panels: scatterplots showing TPM expression. Mean TPM values were calculated for each age group and connected with a line, displayed in ascending order of age groups. The color scale of the line represents the level of significance (false discovery rate [FDR]). Errors bars represent mean ± SEM. (F) Expression levels of downregulated genes related to mitophagy (*PINK1*, *OPTN1*) and mitochondrial quality (*OPA1*). Comparisons and statistical methods as in Figure 1E. For top plot adjusted *p* value is corrected for multiple-testing using the BH method. ∗*p* < 0.05, #*p* < 0.2. Exact *p* values: *PINK1* ICM vs. NF, *p* = 0.004, *PINK1* DCM vs. NF, *p* = 0.00003. *OPTN* ICM vss. NF, *p* < 0.00001, *OPTN* DCM vs. NF, *p* = 0.00003. *OPA1* ICM vss. NF, *p* = 0.008, *OPA1* DCM vs. NF, *p* = 0.17. Lower panel: scatterplots showing TPM expression. Mean TPM values were calculated for each age group and connected with a line, displayed in ascending order of age groups. The color scale of the line represents the level of significance (FDR. Errors bars represent mean ± SEM.

We investigated common signatures that characterize both cardiac aging and heart diseases. The study involved analyzing transcriptomics datasets from the human GTEx study, which included healthy subjects spanning a wide age range.^21^ We compared this with another dataset analyzing transcriptional changes in patients with dilated cardiomyopathy (DCM) and ischemic cardiomyopathy (ICM), compared to non-failing healthy controls (NF).^22^ By applying gene set enrichment analysis (GSEA) and dataset integration, five gene sets were found to be commonly downregulated in both cardiac aging and in disease states (Figure 1A; Table S1). These gene sets encompassed mitochondrial categories, as well as “microtubule,” “nucleoid,” and “organellar ribosome” pathways (Figure 1B). Interestingly, even the latter two gene sets included numerous mitochondrial genes, particularly those involved in mitochondrial transcriptional regulators (Figures S1A and S1B) and mitochondrial translation (Figures S1C and S1D). The pathways “mitochondrial matrix” (Table S1) and “mitochondrial inner membrane” (Figures 1C and 1D; Table S1) exhibited the largest number of enriched core genes across datasets. The most significant downregulation was observed in genes involved in mitochondrial respiration and translation (Figure 1E). Notably, we also observed significant downregulation of several mitophagy genes, including *PINK1* and *OPTN1*, as well as mitochondrial dynamics and cristae regulators, such as *OPA1* (Figure 1F). This indicates that a decline in mitochondrial functions and mitophagy are a common feature of human heart aging and cardiac diseases.

### UA improved cardiac function and mitochondrial health in a model of HFrEF

**Figure 2:**
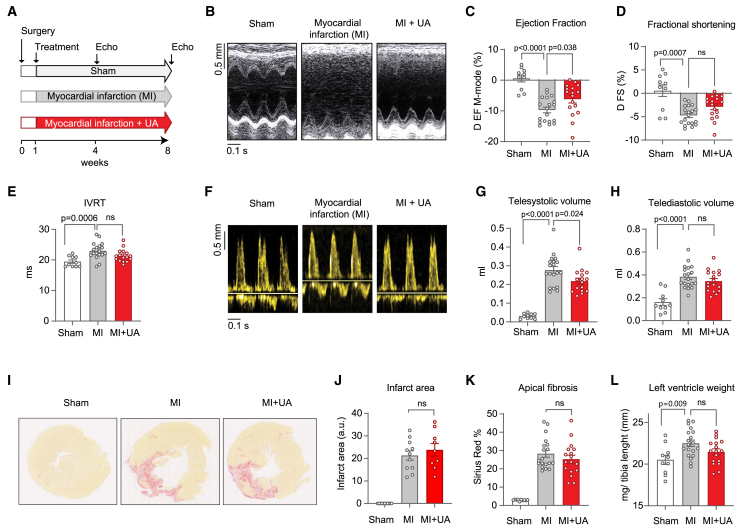
UA protects against cardiac dysfunction in a rat model of heart failure (A) Study schematics of surgery, treatments, and echocardiography timeline for control rats (Sham), rats with myocardial infarction (MI) and MI rats treated with urolithin A (MI + UA). (B) Representative images of M-mode measurement of the left ventricle structure used assess cardiac function and cardiac remodeling. (C and D) Ejection fraction (EF) and fractional shortening (FS) measured as percentage change comparing end of the study to the start of the treatment (1 week time point). ∗*p* < 0.05, ∗∗∗*p* < 0.005 and ∗∗∗∗*p* < 0.001 after one-way ANOVA. Error bars represent mean ± SEM. (E) Measure of the isovolumic relaxation time (IVRT), as aforementioned. ∗∗∗*p* < 0.005 after one-way ANOVA. Error bars represent mean ± SEM. (F) Representative images of echo Doppler for Sham, MI, and MI + UA group used to measure diastolic function. (G and H) Telesystolic volume (G) and telediastolic volume (H) expressed as milliliters in the indicated groups at the end of the study. ∗*p* < 0.05, ∗∗∗*p* < 0.005 and ∗∗∗∗*p* < 0.001 after one-way ANOVA. Error bars represent mean ± SEM. (I–K) Representative images of left ventricle stained with Sirus-RED (I). Corresponding quantification of infarct area expressed as arbitrary units and fold change over sham (J) and of the apical fibrosis percentage expressed as percentage over total LV area (K). (L) Measure of the left ventricle weight expressed as milligrams over tibia length at the end of the study in the indicated groups. ∗∗*p* < 0.01 after Tukey’s multiple comparisons test. Error bars represent mean ± SEM. Number of replicates: Sham (*n* = 10), MI (*n* = 19) and MI + UA (*n* = 17). For (E): Sham (*n* = 10), MI (*n* = 18) and MI + UA (17). For (J) and (K): MI (*n* = 11) and MI + UA (9).

The aforementioned data support the potential to mitigate the progression of heart diseases promoting mitochondrial quality control (QC) pathways. We therefore determined the impact of the mitophagy inducer, UA on a rat model of chronic heart failure with reduced ejection fraction (HFrEF). Myocardial infarction causing reduced systolic function was induced in Wistar Han rats by permanent ligation of the descending coronary artery (MI). A group undergoing sham surgery was used as control (Figure 2A). As expected, MI rats showed a significant decrease in EF one week after surgery, compared to sham (Figure S2A). At this time point, MI animals were randomized based on their EF and administered by gavage with either UA at 25 mpk (MI + UA) or vehicle (MI), for 2 months (Figure 2A).

Echocardiography revealed that UA administration for 2 months improved systolic function in rats with heart failure. The decline of EF and fractional shortening (FS), calculated as the delta between end to beginning of the treatment, was reduced by 35% and 39%, respectively, by comparing UA to vehicle groups (Figures 2B–2D; Table S2). Notably, a trend toward enhanced EF was observed with UA treatment already at the intermediate 1-month time point (Figure S2B). UA also showed a tendency to improve diastolic function, measured by isovolumetric relaxation time (IVRT), despite this being a less striking feature in such a model of chronic heart failure (Figures 2E and 2F; Table S2).

Looking at cardiac remodeling parameters, telesystolic volume was dramatically increased by heart failure and reduced following UA administration (Figure 2G). This supports the beneficial impact of UA on cardiac muscle contractility. Other remodeling readouts, such as telediastolic volume (Figure 2H) remained unchanged in UA versus MI groups. UA did not affect infarct size area (Figures 2I and 2J) and apical ventricle fibrosis (Figure 2K). Finally, a close to significant reduction in the ventricle weight was observed with UA treatment of MI mice (Figure 2L). Altogether, these data indicate a cardioprotective action of UA administration in a model of HFrEF, with major impact of UA on systolic function restoration.

**Figure 3:**
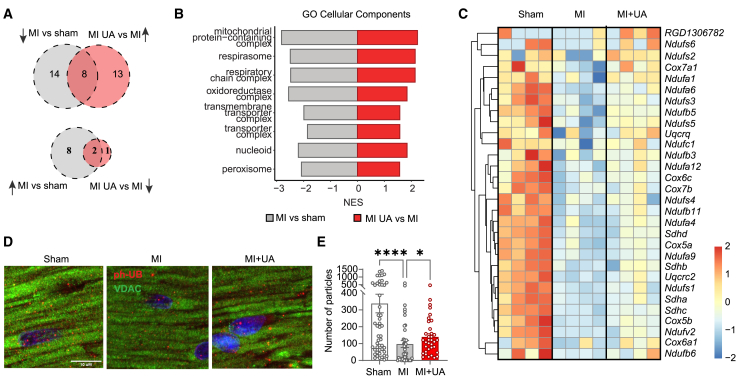
UA rescues defects in mitochondrial gene expression in rats with heart failure (A) Venn diagram depicting overlap of significantly enriched Gene Ontology cellular components (GO CCs) identified by gene set enrichment analysis (GSEA). Top diagram shows GO CCs suppressed in myocardial infarction (MI) versus sham and rescued by UA (“MI UA vs. MI”). Lower panel: similar as aforementioned, depicting GO CCs that are activated in the “MI vs. Sham” comparison and suppressed in the “MI UA vs. MI” comparison. (B) Stacked bar graphs summarizing the 8 common GO CCs depicted in (A, upper panel). The x axis represents the normalized enrichment score (NES), indicating the level of suppression (negative values) or activation (positive values). The “MI vs. Sham” comparison is represented in gray, while the “MI UA vs. MI” comparison is shown in red. (C) Heatmaps displaying scaled normalized expression counts (*Z* scores) of the genes that contributed to the enrichment of the “respiratory chain complex” GO CC term in both “MI vs. Sham” and “MI UA vs. MI” comparisons (respectively suppressed and activated). Rows represent genes and were hierarchically clustered using a Euclidean distance metric and complete linkage. Columns represent individual samples from each condition. (*n* = 4). (D and E) Representative images of left ventricles (LV) stained for VDAC (green) and phospho-ubiquitin (ph-UB, red) in the indicated groups. Nuclei were stained in blue with DAPI. Quantification of the number of particles corresponding to ph-Ub staining (E). (*n* = 54 images/group) ∗*p* = 0.0266; ∗∗∗∗*p* < 0.0001 after one-way ANOVA Kruskal Wallis test. Error bars represent mean ± SEM.

To investigate mechanisms underlying the *in vivo* cardioprotective effects of UA, we performed RNA sequencing (RNA-seq) on heart sections from rats described previously. A GSEA identified pathways that were significantly altered with HF and rescued by UA treatment, the majority of which were downregulated with HF and then increased by UA (Figure 3A; Table S3). They included several pathways associated with mitochondrial respiration, as well as nucleoid and peroxisome biology (Figure 3B). A decline in mitochondrial gene sets in this rat model is consistent with data from human subjects (Figures 1B–1D). Several genes whose expression is rescued by UA in diseased animals are related to mitochondrial oxidative phosporylation (OXPHOS), complexes (Figures 3C and S2C). No changes were observed for mitophagy related genes in this setting. We further measured mitophagy at the protein levels, in heart sections from the MI animals with or without UA treatment. Consistent with previous studies in other age-related conditions,^16^^,^^20^^,^^23^ UA was able increase levels of the PINK1/parkin-mediated mitophagy marker, phospho-ubiquitin (ph-Ub), in the heart of MI animals, compared to vehicle (Figures 3D and 3E).

### UA improved cardiac and skeletal muscle function in old mice

**Figure 4:**
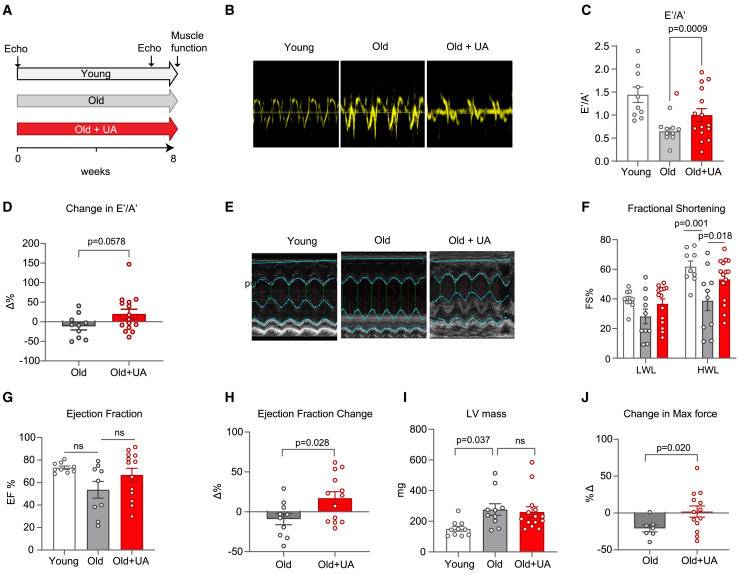
UA improves aging cardiac function and skeletal muscle force (A) Study schematics of both young and old mice feeding and experimental timeline. (B) Representative image of echo Doppler flow measure used to assess diastolic function. (C and D) Diastolic function of blood flow across the mitral valve in early diastole (Ea/E′) to the flow in late diastole (Aa/A′). Diastolic function represents early to late diastole ratio (E′/A′) in young and aged mice (C) and change in E′/A′ (post-treatment–baseline) in aged mice (D) ∗∗∗∗*p* < 0.001 after Welch t test. Error bars represent mean ± SEM. (E) Representative image of M mode short axis to assess left ventricle. ∗∗∗*p* < 0.005 after one-way ANOVA. Error bars represent mean ± SEM. (F) Fractional shortening (FS%) under high workload (HWL) and low workload (LWL). ∗*p* < 0.05 and ∗∗∗∗*p* < 0.001 after two-way ANOVA. Error bars represent mean ± SEM. (G and H) Ejection fraction at endpoint (G) and change in ejection fraction (H) in aged animals. *p* value = ns after one-way ANOVA. Error bars represent mean ± SEM. ∗*p* < 0.05 after t test. Error bars represent mean ± SEM. (I) Left ventricle mass from heart weights after sacrifice expressed in milligrams. ∗*p* < 0.05 after one way ANOVA. Error bars represent mean ± SEM. (J) Maximal torque of plantar flexor by nerve stimulation expressed as changes over baseline. ∗*p* < 0.05 after t test. Error bars represent mean ± SEM. Animal replicates: cardiac data: young = 9, old = 10, old + UA = 15; skeletal muscle data: old = 7, old + UA = 13.

We then investigated for the first time the impact of UA on supporting cardiac function in non-diseased, old rodents. Cardiac measurements were performed in 24-months old male C57BL/6 mice fed with UA or standard chow diet for 2 months, using 5-month-old young mice as controls. (Figure 4A). To assess diastolic function, the ratio of blood flow across the mitral valve in early diastole (Ea/E′) to the flow in late diastole (Aa/A′) was determined. This was done using echocardiographic imaging, as illustrated in Figure 4B. A comparison between old and young mice revealed a reduced ratio of early to late diastolic mitral annulus velocities (E′/A′) in the aged mice, indicating a decline in diastolic function. Notably, the treatment of UA significantly preserved the age-related decline in diastolic function when compared to the old control group at the end of the 8-week treatment period (Figure 4C). When looking at the delta of E′/A′, comparing end of the study to baseline, the comparison between old UA and old control mice approached significance (*p* = 0.0578) (Figure 4D). To assess systolic function, the percentage of FS was measured using echocardiography’s M-mode short axis. Anesthetized mice were subjected to lower-than-physiological heart rates, which may not accurately represent systolic function under compromised low workload (LWL) conditions. Therefore, FS was also measured following the injection of the inotropic agent dobutamine to induce a higher heart rate within the physiological range (Figure 4E). There was no significant difference in FS between groups at the LWL (Figure 4F). However, under higher workload (HWL) conditions, UA treatment rescued the decline in FS associated with aging (Figure 4F). Furthermore, a trend toward the preservation of EF was observed after 8 weeks of UA feeding (Figure 4G). These changes in EF were statistically significant when analyzed as percentage comparing end of the study to baseline between the old UA-treated and old groups (Figure 4H). Increased left ventricular mass (LVM) was observed with aging and suggests cardiac hypertrophy, either due to increased functional demand or pathological conditions specific to the heart or in combination with other organs. UA treatment only mildly affected LV mass hypotrophy phenotype in aged mice (Figure 4I). Functional changes in skeletal muscle were assessed after 8 weeks of UA feeding. The maximal torque of the plantar flexors was tested comparing end of the experiment to baseline for both control and UA-treated old mice. The UA group maintained maximal force compared to baseline, an effect that was significantly different when compared to the decline over time observed in the old control group (Figure 4J). We also tested UA treatment on heart mitochondria respiration by oxygraphy. No difference was detected between the old controls and the old UA-supplemented group (Figures S3A–S3C).

### UA improves heart muscle mitochondrial morphology and ultrastructure

In a parallel study of natural aging, we treated 21-month and 2-month-old C57BL/6Rj mice with UA for 8 weeks. We collected heart tissues for young and old UA-treated mice for further phenotyping of mitochondrial functions and activities,^24^ such as mitochondrial dynamics, cristae remodeling and mitophagy.

**Figure 5:**
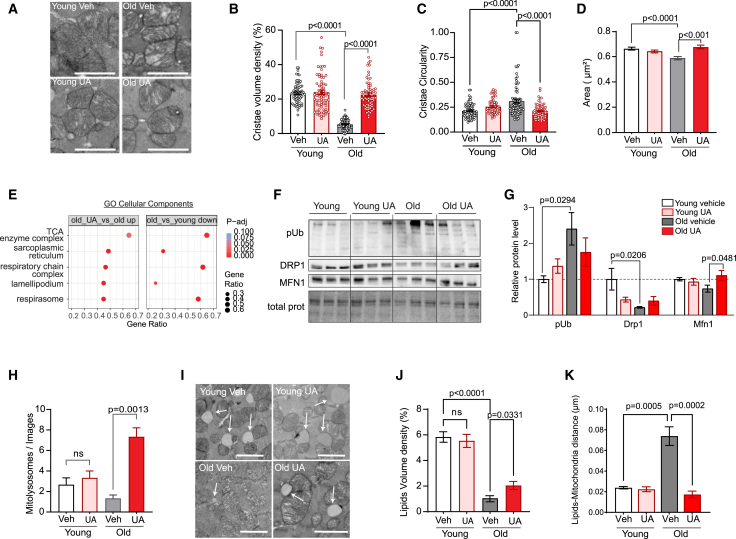
UA improves heart mitochondrial ultrastructure, morphology and function in old mice (A–C) Representative heart transmission electron microscopy (TEM) of transversal images of young and old mice administered with either vehicle or UA for 2 months (A) and corresponding quantification of mitochondrial cristae density (B) and circularity (C). *n* = 75 mitochondria/group. ∗∗∗∗*p* < 0.001 after one-way ANOVA. Error bars represent mean ± SEM. Scale bars: 500 nm. (D) Quantification of mitochondrial area in transversal orientation in young and old mice from vehicle and UA groups (*n* = 3 animal/group). ∗∗∗*p* < 0.005 after one-way ANOVA. Error bars represent mean ± SEM. (E) Gene set enrichment analysis from house heart aging RNA-seq data describing Gene Ontology (GO) cellular component gene sets that are significantly repressed with cardiac aging (old vs. young down) and significantly rescued by UA administration to old mice (old UA vs. old up). The size of each dot corresponds to the gene ratio, while the color indicates the statistical significance of the enrichment (Benjamini-Hochberg-adj. *p* value). (F and G) Representatives immunoblot of phospho-ubiquitin, DRP1, MFN1 and stain free blot of total proteins (F) and quantification of phospho-ubiquitin, DRP1, and MFN1 (G) protein content normalized over their corresponding total protein stain free blot (*N* = 6 animal/group). *p* value. ∗*p* < 0.05 after one-way ANOVA. Error bars represent mean ± SEM. (H) Mitolysosome events quantification in young and old mice treated as indicated (*n* = 3 animal/group). ∗∗∗∗*p* < 0.001 after one-way ANOVA. Error bars represent mean ± SEM. (I–K) Representative images with arrows pointing to lipid droplets (I) and their quantification of volume density expressed as percentage over total area in the indicated animals (J) and quantification of the distances between lipids and mitochondria (*n* = 3 animal/group) on the two-dimensional electron micrographs (K). *p* value. ∗∗∗*p* < 0.005; ∗∗∗∗*p* < 0.001 after one-way ANOVA. Error bars represent mean ± SEM. Scale bars: 500 nm.

First, we used transmission electron microscopy (TEM) to measure the mitochondrial ultrastructure (cristae), content, and morphology. Mitochondrial cristae density was markedly decreased with age, as evidenced by the loss of strict lamellar position (Figures 5A and 5B). UA intervention rescued the age-dependent decline in cristae volume density to levels compared to those observed in young animals (Figures 5A and 5B). Interestingly, mitochondrial cristae were more circular with age that may reflect aberrant mitochondrial remodeling in response to cellular stress and metabolic alterations, while UA administration to old animals reverted such increase (Figure 5C).

Regarding mitochondrial morphology, mitochondria appeared to be smaller in old compared to young animals, an effect rescued by UA administration (Figure 5D). No changes in mitochondrial volume density and number were observed with UA in either young or old mice (Figures S4A and S4B). These results indicate that age-associated alteration in heart mitochondrial morphology and ultrastructure are reversed by 2 months of UA supplementation, while neither aging nor UA affected mitochondrial content.

Heart RNA-seq in these animals was performed to investigate whether these mitochondrial changes were driven by a transcriptional program. An unbiased ranking of top age-related pathways declining with aging and rescued by UA identified genes and pathways mostly related to mitochondria tricarboxylic acid (TCA), cycle (Figures 5E; Table S4).

When looking at mitochondrial dynamics, immunoblotting analysis revealed an alteration of mitochondrial dynamics proteins DRP1 and MFN1 with aging, and the rescue of their levels by UA (Figures 5F and 5G). In addition, we observed an accumulation of mitophagy markers, phospho-Ub (Figures 5F and 5G) and OPTN in aged hearts, which were partially normalized with UA (Figures S4C and S4D). This indicates that aged mitochondria decorated with mitophagy receptor are stalled in this stage, suggesting a defect in mitophagosome formation. UA was indeed shown to promote lysosomal function and through increasing the structural component LAMP1.^25^ Further immunoblotting in our study showed a reduction of LAMP-1 with aging with its levels rescued with UA, supporting UA role in reverting stalled mitophagy with aging through lysosomal activation (Figures S4C and S4D). To confirm these findings, the number of mitolysosomes was quantified by TEM. Consistently with the aforementioned data, aged mice showed a significant reduction in mitolysosome structures in the heart while UA supplementation significantly rescued in the number of mitophagy events (Figures 5H and S4E).

Finally, we quantified the volume density of heart lipid droplets and identified a reduction in old compared to young animals, and significant rescue in old mice treated with UA (Figures 5H and 5J). In addition, the distance between mitochondria and lipid increased with aging and was recovered with UA (Figure 5K), suggesting that UA administration may help maintain lipid homeostasis and support the mitochondria-lipid interactions in the aging heart.

These results indicate that 2 months of UA administration activate mitochondria recycling and quality control to achieve a mitochondrial pool with improved quality.

### UA reduces plasma ceramides associated with CVD risk in mice and humans

We investigated the translational potential of these findings for humans by assessing UA’s impact on clinically relevant cardiac health biomarkers. Plasma sphingolipids that play a key function in heart disease development and emerged as new plasma biomarkers predicting CVD risks^26^^,^^27^ were quantified by liquid chromatography-tandem mass spectrometry (LC-MS/MS).

**Figure 6:**
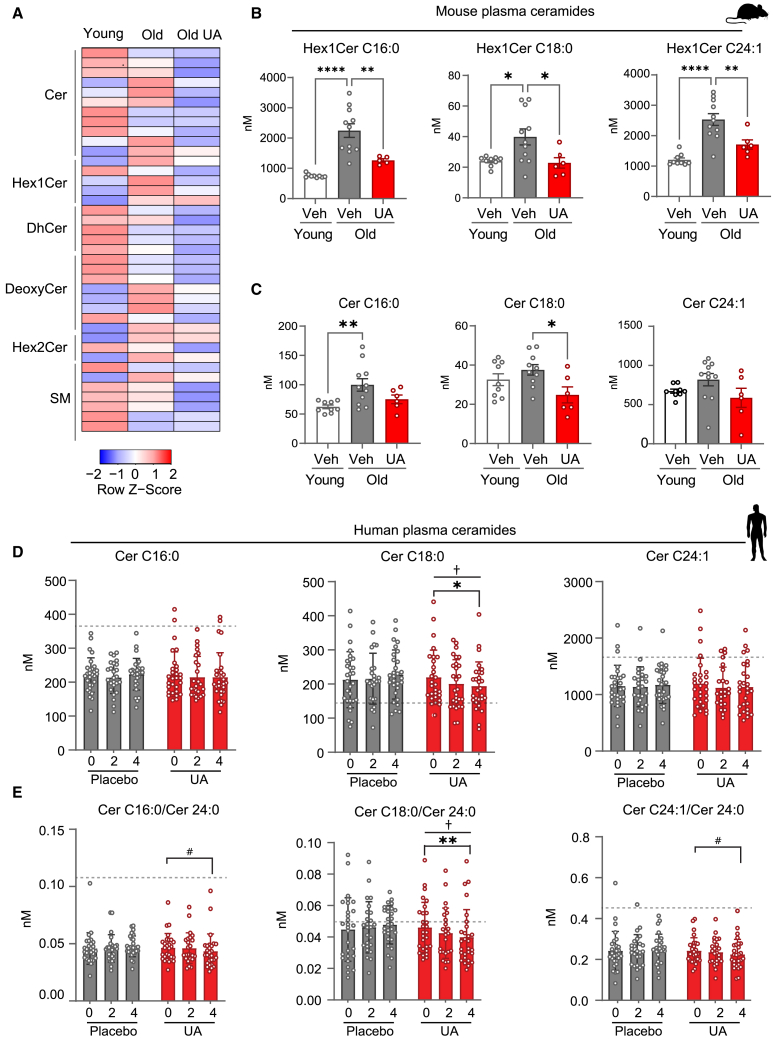
UA lowers plasma ceramides linked to increased CVD risk in mice and humans (A) Heatmap representing the concentration of different sphingolipid species in the plasma of mice. Values represent row *Z* scores. Cer, ceramides; Hex1Cer, hexosyl-1-ceramides; DhCer, dihydroceramides; Deoxycer, deoxyceramides; Hex2Cer, hexosyl-2-ceramides; SM, sphingomyelins. (B and C) Concentrations of Hex1Cer, C16:0, C18:1, and C24:1 (B) and ceramides C16:0, C18:0, and C24:1 (C) in plasma of the indicated mice groups. *p* value. ∗*p* < 0.05; ∗∗*p* < 0.01; ∗∗∗∗*p* < 0.001 after one-way ANOVA. Error bars represent mean ± SEM. Number of animals: young vehicle (*n* = 9), old vehicle (*n* = 11), and old UA (*n* = 6). Detailed statistical information indicating exact *p* values and fold change values in Table S5. (D) Concentrations of ceramides C16:0, C18:0, and C24:1 (C) in plasma of healthy elderly subjects supplemented with either placebo or urolithin A (1 g) at baseline (0), after 2 months or 4 months ^#^*p* < 0.1, ∗*p* < 0.05, ∗∗*p* < 0.01, ANCOVA comparing single time point over baseline and within treatment groups; ^†^*p* < 0.05 ANCOVA, visit effect (baseline vs. 2 months vs. 4 months). (E) Ratio of plasma concentrations of ceramides C16:0, C18:0, and C24:1 as in (C) over the plasma concentration of the housekeeping ceramide C24:0. ^#^*p* < 0.1, ∗*p* < 0.05, ∗∗*p* < 0.01, ANCOVA comparing single time point over baseline and within treatment groups; ^†^*p* < 0.05 ANCOVA, visit effect (baseline vs. 2 months vs. 4 months) and within treatment groups. Error bars represent mean ± SEM. Number of replicates: placebo baseline (*n* = 29), placebo 2 months (*n* = 25), and placebo 4 months (*n* = 29). UA baseline (*n* = 29), placebo 2 months (*n* = 25), and placebo 4 months (*n* = 29). Detailed statistical information indicating exact *p* values and fold change values in Table S5.

In mice, aging induced a complex remodeling of the sphingolipid plasma profile, while UA treatment led to an overall decrease of most species (Figure 6A). Sphingolipids whose levels were most significantly rescued by UA in aging mice were enriched for hexosyl-ceramides (Table S5; Figure 6B) and N-nervonoyl species (Table S5; Figure S5A).

Notably, aging induced a significant increase in the ceramide species, C16:0 and C24:1 that, together with C18:0, are part of the ceramide risk score (CERT1, ceramide test 1), a clinically validated panel used to predict risks of CVDs (Figure 6C).^28^ UA supplementation led to a significant or trending reduction of deleterious C16:0, C18:0, and C24:1 (Figure 6C), indicating that the improvement of heart functions is reflected in the systemic biomarker profile of heart health.

To translate these findings in humans, the same sphingolipid analysis was performed in plasma from a randomized, placebo-controlled study described previously,^19^ in which healthy older adults were administered placebo or 1 g/day of UA (NCT03283462). Samples were collected at baseline, and after 2 months and 4 months of supplementation. We assessed changes by UA over time, controlled over changes in the placebo group. The analysis showed a robust impact of UA on human plasma sphingolipids, with major reduction in middle and long-chain hexosy-1-ceramides and ceramide species (Figures S5B; Table S6). Importantly, ceramide species part of the CERT1 score were significantly impacted by UA, especially the deleterious ceramides whose accumulation was found to be significantly positively associated with CVD risk, i.e., ceramides C18:0 and C24:1 (Figure 6D). UA-mediated improvement was further confirmed after calculating the ratios of C16:0, C18:0, and C24:1 to the most abundant circulating specie, ceramide C24:0 (Figure 6E).^28^ These ratios are also part of the CERT score and C24:0 is used since it is considered to be benign and with no association with CVD.^28^

## Discussion

Aging is a major risk factor of heart diseases, the world’s main cause of death causing 17.9 million deaths worldwide.^29^ Exercise and heart-healthy dietary patterns are part of the guidelines to improve cardiovascular health as we age.^30^ In addition, nutrient supplements have potential for further benefits, considering that adhering to heart-healthy diets presents substantial challenges.^30^ Omega-3 is among the most studied nutritional intervention in this context. Clinical studies showed efficacy in subjects with high risk of CVD,^31^ but conflicting results in general populations at usual risks.^32^ Mechanisms underlying the potential benefits of omega-3 for heart health includes lowering triglycerides and increasing healthy high-density lipoprotein levels.^33^ Other clinical studies with nutritional interventions tested for hearth health have yielded negative results.^30^ Today there is a lack of nutritional interventions that are effective and target key drivers of cardiac cell health before onset of heart diseases.

Here, we identify mitochondrial dysfunction as a fundamental hallmark of human heart aging and disease, and UA as a nutritional intervention able to rescue mitochondrial quality inside heart cells and improve heart functions in preclinical models of heart aging and disease. Finally, we translated the potential of UA for human heart health showing results from a clinical study where UA significantly reduced clinically validated ceramide species associated with risk of CVD found in the plasma.

Mitochondrial dysfunction is one of the twelve hallmarks of aging,^34^ and was shown to be a mechanistic driver of heart failure in multiple pre-clinical models.^35^^,^^36^^,^^37^ Our gene expression analysis in human heart biopsies confirmed that mitochondrial dysfunction is a common hallmark of both heart aging and cardiomyopathies in humans. We identified genes associated with oxidative phosphorylation, mitochondrial QC, and mitophagy, among the most prominent transcriptional responses to aging, dilated and ICM. This intersection points to the translational relevance of improving mitochondrial QC to protect against the onset and progression of heart dysfunction in humans.

A decline in mitochondrial QC in the aging heart was described by the disruption in mitochondrial cristae structure in several preclinical reports.^38^^,^^39^ Using EM, we confirmed this evidence in old mice and showed that their administration of UA for 8-weeks reversed age-related changes in mitochondria ultrastructure and mitophagy. We also revealed a marked lipid droplet volume density depletion in cardiac aging and a significant replenishment after UA supplementation. This might reflect a crosstalk and energy transfer between lipid droplets and mitochondria.^40^

At the functional level, UA significantly counteracted heart dysfunction. This was observed in preclinical models of both cardiac aging and heart failure with EF. Previous work has demonstrated that enhancing mitophagy by treating with UA reduces cardiac dysfunction in models of diabetes,^23^^,^^41^ obesity,^42^ and sepsis.^43^ The present report is the first investigation of the beneficial effects of UA on the aging heart and the successful translation of these findings in humans, using clinically validated biomarkers.

A key feature of the aging human heart is diastolic dysfunction, or heart failure with preserved ejection fraction (HFpEF).^44^^,^^45^^,^^46^ As a result, many previous preclinical studies with mitochondrial interventions focused primarily on improvements in diastolic dysfunction.^47^^,^^48^^,^^49^ In this study we observed an improvement of both systolic EF and FS, as well as a partial correction of diastolic deficits in the aging mouse heart following UA treatment. Effects were enhanced by stimulating cardiac work with dobutamine to a more physiologically relevant workload. These results make UA unique among other nutritional interventions targeting mitochondrial for the aging heart, in that UA improves both diastolic and systolic function in the models investigated. Indeed, other nutritional interventions like spermidine were reported to improve only diastolic dysfunction,^48^ while interventions targeting NAD+ metabolism like NMN improved only systolic dysfunction.^50^ Among pharmacological approaches, elamipretide was reported to improve diastolic dysfunction in the aging heart,^47^ and more recent work demonstrated effects on systolic function as well.^51^

Despite the focus on HFpEF with age, many age-related changes in the heart increase the susceptibility of the aging heart to heart failure with reduced EF (HFrEF). We show the ability of UA supplementation to improve mitochondrial and cardiac function in a rat myocardial ischemia model of chronic heart failure. In this model, UA significantly improved systolic function and rescued gene expression changes observed in diseased rats after 8-week of administration.^52^

Our results have significant clinical potential. Indeed, UA has already been tested in clinical studies with proven safety and positive clinical endpoints related to improved muscle function. In older adults two months of supplementation led to increased muscle endurance.^19^ In middle aged overweight subjects, 4 months of UA supplementation led to significant increase in muscle strength and a trend to increase of peak VO2 by 10%.^20^ In addition, we highlight that UA supplementation showed maintenance of skeletal muscle force in aged mice. This is clinically relevant as skeletal muscle mitochondrial function is impaired in patients with HFpEF and contributes to the reduced exercise tolerance and quality of life.

Analysis of the plasma sphingolipids in adults taking UA further highlights the clinical relevance of UA supplementation in humans. In healthy older adults, UA significantly reduced plasma ceramides that are part of the CERT, a clinically validated ceramide score, that has been strongly associated with CVD risk in different risk categories. This score has been proposed as a more sensitive and accurate marker compared to cholesterol, with potential to replace the latter in the future.^28^^,^^53^ The CERT score is also linked to insulin resistance and diabetes that are today known to be diabetogenic and atherogenic when they accumulate over longer periods of time.^53^

Together with previous results from clinical trials demonstrating improved skeletal muscle function, these data point to UA supplementation as a new approach targeting cellular mitochondrial health to enhance healthy heart aging and quality of life.

### Limitations of the study

The main limitation of this study is that data from the aging heart were derived from two parallel studies with different UA delivery methods. In one study, a chow diet was employed to assess its benefits measured with *in vivo* cardiac functional assays, and in a second study, daily oral gavage was employed to assess impact on mitochondrial ultrastructure and gene expression. Ideally, both mitochondria ultrastructure and *in vivo* function would have been measured in a single study. Despite differing dosing strategies and endpoints, both methods significantly reversed aspects of cardiac aging, indicating a robust effect of UA. Moreover, while the mouse aging model partially resembles diastolic dysfunction present in HFpEF, a more precise model, such as the LNAME+ high fat diet^54^ would be relevant for further validation. Finally, the heart failure data do not address UA’s impact on acute HF that would require additional studies in ischemia-reperfusion models.

## Resource availability

### Lead contact

Further information and requests for resources and reagents should be directed to and will be fulfilled by the lead contact, Davide D’Amico (ddamico@amazentis.com).

### Materials availability

This study did not generate new unique reagents.

### Data and code availability

• RNA-seq data that support the findings of this study will be deposited in the NCBI Gene Expression Omnibus (GEO; http://www.ncbi.nlm.nih.gov/geo/). Mouse aging study GEO: GSE283273. Rat heart failure study - GEO: GSE283003.
• This paper does not report original code
• Any additional information required to reanalyze the data reported in this paper is available from the lead contact upon request.

## Acknowledgments

We thank Leonidas Karagounis Global Science Lead, Muscle Metabolism & Metabolic Health at Nestlé Heath Science during the course of this study and now Professor of Research Translation & Enterprise Professor of Research Translation & Enterprise at the 10.13039/501100000990Australian Catholic University for the support in his critical comments during the design of the studies. We also thank Tony Teav and Rebecca Borreggine at the Metabolomics Platform at UNIL for their contribution with sphingolipid analysis.

## Author contributions

D.D., D.J.M., A.S., C.R., and P.A.A. contributed to the design of the study. J.F. coordinated the cardiac aging study and collected samples for EM (study 1). R.L. and T.D. performed EM imaging. J.F. and C.T. analyzed EM images and interpreted data. S.L. performed the cardiac aging study 1 and interpreted all data; G.B. performed the rat heart failure study. C.T. performed all immunofluorescence analyses and interpreted these data. C.T. performed sample preparation for RNA-seq analyses. D.K. performed all bioinformatic analyses. J.I. and H.G.-A. contributed to sphingolipid analyses. D.D. and D.J.M. wrote the manuscript, with the help of the other co-authors. All authors reviewed the manuscript.

## Declaration of interests

J.F., C.T., D.D., A.S., and C.R. are currently employees and P.A.A. was a past employee of Amazentis SA, which holds patents on UA applications. S.L. reported receiving drugs at no cost for research use from Stealth Biotherapeutics and supplements at no cost for research use from AstaReal outside the submitted work. D.J.M. reported receiving drugs at no cost for research use from Stealth Biotherapeutics, grants from Boehringer Ingelheim, and supplements at no cost for research use from AstaReal outside the submitted work. This research was supported by Amazentis SA and D.J.M. received support from NIH P01 AG001751.

## STAR★Methods

### Key resources table

**Table:** 

REAGENT or RESOURCE	SOURCE	IDENTIFIER
**Antibodies**		

Anti-phospho ubiquitin	Millipore	Cat# ABS1513-I; RRID: AB_2858191
anti-OPTN	Proteintech	Cat# 10837-1-AP; RRID:AB_2156665
anti-LAMP1	Abcam	Cat# ab24170; RRID:AB_775978
anti-DRP1	Cell Signaling	Cat# 8570; RRID:AB_10950498
anti-MFN1	Proteintech	Cat# 13798-1-AP; RRID: AB_2266318
Rabbit Anti-Mouse IgG H&L (HRP)	Abcam	Cat# ab6728; RRID: AB_955440
Goat Anti-Rabbit IgG H&L (HRP)	Abcam	Cat# ab6721; RRID: AB_955447
Anti-VDAC	Abcam	Cat# ab14734; RRID:AB_443084
Goat Anti-Rabbit IgG (H+L) Antibody, Alexa Fluor 555 Conjugated	Invitrogen	Cat# A-21428; RRID:AB_141784
Goat anti-Mouse IgG (H+L) Cross-Adsorbed Secondary Antibody, Alexa Fluor™ 488	Invitrogen	Cat# A-11001; RRID:AB_2534069

**Chemicals, peptides, and recombinant proteins**		

Protease inhibitor	Thermofisher	Cat# 78430
Phosphatase inhibitor	Thermofisher	Cat# 78428
Laemmli buffer	Biorad	Cat# 1610747
Tris-Buffered Saline containing 0.1% Tween 20	Brunshwig	Cat# SER42598-01
Clarity ECL substrate	Biorad	Cat# 170–5,060
Qiazol	Qiagen	Cat# 79306
deparaffinization solution	Qiagen	Cat# 19093
Triton X-100	Sigma	T8787
Bovine Serum Albumin	Pan biotech	Cat# P06-1391100
Goat serum	Jackson Immuno Research	Cat# 005-000-121
Vectashield Antifade Mounting Medium	Adipogen	Cat# H-1500
Urolithin A	Timeline	N/A
Acetone	Fisher	A060617
Cacodylate buffer	Agar Scientific	Cat# R1104
Copper grids	Gilder grids	GA 1500-C3
Epoxy resin	TAAB	Cat# T030
Glutaraldehyde	TAAB	Cat# G003
Osmium tetroxide	Agar scientific	AGR1024
Toluidine blue	TAAB	Cat# SD211
Lead Citrate	Sigma-Aldrich	Cat# 228621
Uranyl acetate	Agar Scientific	Cat# AGR1260A

**Criticalcommercial assays**		

RNeasy FFPE Kit	Qiagen	Cat# 73504
DC protein assay	Biorad	Cat# 500-0112

**Deposited data**		

Raw and analyzed data – Mouse aging study	This paper	GEO: GSE283273
Raw and analyzed data – Rat heart failure study	This paper	GEO: GSE283003

**Experimental models: Organisms/strains**		

Mouse: C57BL/6J wild type	NIA	N/A
Mouse: C57BL/6Rj wild type	Janvier Labs	N/A
Rat: Wistar male	Janvier Labs	N/A

**Software and algorithms**		

GraphPad Prism 9	GraphPad	https://www.graphpad.com/scientific-software/prism/ version 9
ImageJ	NIH	version1.52 https://imagej.nih.gov/ij/
ImageLab	Bio-Rad	version 6.1
Clinical Trace Finder software	Thermo Fisher Scientific	version 4.1
fastP		version 0.23.2
STAR aligner		version 2.6.1c
R package biomaRT		v. 2.46.3
MultiQC		version 1.8.dev0
R VennDiagram package		
R package ggplot2		
DESeq2		
R package ClusterProfiler		

**Other**		

15% Mini-PROTEAN™ TGX Stain-Free™ Protein Gel stain-free	Biorad	4568086
Transblot transfer	Biorad	1704156
Diets (mouse aging study 1)	Research diets	AIN93G
Diets (mouse aging study 2)	SafeLab	irradiated pellet A04
Diet (rat heart failure study)	SDS diet	
Echocardiography	GE Medical Systems	Vivid 7
Echocardiography	Vevo Imaging Station	Vevo 3100 preclinical imaging system
Pelco BioWave Pro+ Microwave Processing System	Ted Pella	N/A
HT 7800 TEM	Hitachi	N/A
AMT 40 CCD Camera	Deben	N/A

### Experimental model and study participant details

#### Cardiac aging animal studies

For cardiac phenotyping study (Study 1), all animal experiments were approved by the University of Washington Institutional Animal Care and Use Committee. A total of 40 aged (24–26 months) C57BL/6J mice and 10 young (5 months) male mice were received from NIA. All mice were maintained at 21°C and a 14/10 dark cycle at 30–70% humidity feeding standard chow (LabDiet PicoLab Rodent Diet 20) and water *ad libitum* before the experimental diet was administered. Animals were fed with control diet (Research Diets AIN93G) or diet supplemented with UA oral targeting 50 mg/kg/day for 8 weeks.

For the mitochondrial morphology and activity animal study (Study 2), animal experiments were performed in accordance to European guidelines for care and were approved by the Animal Ethical Committee, the Higher Education and Research Ministry. Twenty-four male C57BL/6Rj mice aged 21 months old and eighteen male C57BL/6Rj mice aged 8 weeks old were purchased from Janvier Labs (France). Mice were collectively housed in cages in controlled room at 22°C and 12 h light/dark cycle at Biomeostasis’ facilities. All animals were allowed ad libitium access to an irradiated normal chow diet (NC, irradiated pellet A04; SAFE, Villemoisson-sur-Orge, France) and to ultrapure and laboratory-grade acidified water (Aquavive, INNOVIVE, France). For 8 weeks, 21-months-old and 8-week-old mice were treated by oral gavage, either with a vehicle solution (0.5% carboxymethylcellulose) (21 months vehicle, *n* = 12 or 8 weeks vehicle *n* = 9) or with UA at 50 mg/ml (21 months UA, *n* = 12 or 8 weeks UA, *n* = 9). One day after the last dosing, overnight-fasted mice were anesthetized by Isoflurane. The hearts were collected, weighed and the LV and RV were separated. One part of LV was embedded in OCT following the protocol described in previous study.^55^ The other parts of the LV were collected for EM. RV were snap frozen.

#### Heart failure animal study

The experimental procedures were carried out in accordance with European guidelines for the care and use of laboratory animals (Directive 2010/63/UE). The protocol that was used to induce myocardial infarction (MI) in rats was approved by an Animal Ethical Committee (French National Committee N°71) and by the Higher Education and Research Ministry (#30207–2021021117467048 v4) on April 11th, 2021. Fifty Wistar male rats were purchased from Janvier Labs (France). Animals were collectively housed in cages at SYNCOROSOME’s facilities and had free access to food (RM1, SDS Dietex) and drinking water *ad libitium.* Surgery was initiated in 7-week-old rats that were separated into three groups. MI was induced in animals from Surgery Vehicle (*n* = 20) and Surgery UA (*n* = 20), by chronic left anterior descending coronary artery (LAD) ligation performed at day 0. Rats were anesthetized by an intraperitoneal injection (IP) of Medetomidine (0.5 mg/kg) and Ketamine (50 mg/kg). The animals were then intubated and ventilated at 10 mL/kg tidal volume and 70–80 cycles/minutes. The body temperature was maintained around 37°C using a thermoregulated heating pad connected to a rectal probe. Rats were placed in supine position, chest-shaved and prepared for standard surgical aseptic conditions. A left lateral thoracotomy was carefully performed to expose the heart. Suture was placed around the LAD (4/0 Silk, Ethicon), around 2 mm below the left atrium and close to the interventricular junction, to obtain infarct size (IS) near 40% of the total left ventricular (LV) area. The chest was closed, air was expelled from the rib cage to avoid pneumothorax and quick reanimation was performed using atipamezole hydrochloride (IM, 0.5 mg/mL, Zoetis).

The sham vehicle animals (*n* = 10) were subjected to the same protocol. However, after the left lateral thoracotomy exposing the heart, the rib cage was closed without passing the suture thread around LAD. A peri-operative care of the animals was performed during the surgery.

For 8 weeks, starting 24 h after the surgery UA animals were daily treated with UA at 50 mg/mL by oral gavage. The rats from sham vehicle and surgery vehicle group received the same dose of a vehicle solution (0.5% carboxymethylcellulose) at the same time. The volume of administration was adjusted every week based on the mean body weight.

#### Study participants of sphingolipid analysis

Sphingolipid analysis was performed in plasma from a randomized, placebo-controlled study described previously (Energize study, NCT03283462).^19^ Subjects were screened on physical performance (6-min walk distance ≤550 m) and low-average mitochondrial health using MRS that evaluated the maximal ATP synthesis rate (≤1.0 mM/s) in the hand skeletal muscle (first dorsal interosseus [FDI]). The recruited participants had a mean (SD) age of 71.7 (4.94) years, with 50 women (75.8%) and 16 men (24.2%); and were all White individuals.

Healthy older adults were administered placebo or 1g/day of UA. Plasma samples for sphingolipid analysis were collected at baseline, and after 2 months and 4 months of supplementation.

### Method details

#### Human heart failure and GTEX RNA-seq data

Data from a human heart failure study were downloaded from the GEO database under the accession number GSE116250, using the fastq-dump command from the SRA toolkit. The data were downloaded in the form of single-end FastQ files, with 64 FastQ files corresponding to 64 samples. The data are summarized under three conditions: Non-failing (NF) with 14 samples, DCM with 37 samples, and ICM with 13 samples. Heart data from GTEX were downloaded from the supplemental material of the study by Yang et al.,^56^ including information regarding upregulated and downregulated genes during aging, which correspond to positive and negative values of the "age" coefficient in the applied regression model, respectively. Additionally, normalized expression values, represented as Transcripts Per Million (TPM), for the respective RNA-seq samples (432 individuals across 54,592 genes) were obtained from the GTEx Consortium Portal (https://gtexportal.org/home/datasets). The dataset encapsulates six distinct age groups: 20–29 (22 samples), 30–39 (26 samples), 40–49 (66 samples), 50–59 (154 samples), 60–69 (150 samples), and 70–79 (14 samples). Data analysis was conducted using a linear regression model to detect aging-related genes. Genes were categorized as either "upregulated" or "downregulated" based on the sign of the "age" coefficient in the corresponding regression model (denoted as "Age coef"). In this study, this dataset is referred to as the "GTEx aging" dataset."

#### Differential expression analysis of human heart failure data

Differential expression analysis was applied between “Ischemic Cardiomyopathy “(ICM) and “Non-failing” (NF) conditions (referred as “ICM_vs_NF”), and between “Dilated Cardiomyopathy” (DCM) and “NF” conditions, using “NF” as reference dataset (referred as “DCM_vs_NF”).

Before the DE analysis, a pre-filtering step was applied to remove low abundance mRNA measurements, by excluding genes with less than 10 total counts across samples. Additionally, an independent filtering procedure of DESeq2 was enabled, in order to filter out genes with very low counts that are unlikely to show significant alterations in gene expression. Gene symbols (MGI nomenclature), descriptions, and biotypes were matched to Ensembl GTF ids by using the R package biomaRt. v. 2.46.3. *p*-values were corrected for multiple-testing using the BH method.^57^

#### Gene set enrichment analysis – Human heart aging and heart failure datasets

The database used for GSEA enrichment was the Cellular Components collection of the Gene Ontology database (GO CCs). The enrichment analysis for the human heart aging (GTEx aging) and for each heart failure comparison (“ICM_vs_NF” and “DCM_vs_NF”) was performed separately, using the R package ClusterProfiler.

For the GTEx aging dataset, the age coefficient (“Age Coef”),^56^ was used as a gene ranking metric, while for each heart failure comparison, genes were ranked by the respective log2 fold change value. Consequently, the enrichment of GO CC gene sets among up- or-down regulated genes was statistically tested. The minimum and maximum gene set sizes were set to 10 and 500, respectively. The resulting *p*-values were adjusted using the BH method.

Terms with an adjusted *p*-value ≤0.05 were considered as statistically significant and were further characterized as activated or supressed based on the respective NES sign (activated for NES >0 and supressed for NES <0). Common and unique repressed GSEA GO CCs between heart aging and heart failure datasets were visualized as a Venn diagram, using the R VennDiagram package. Common repressed terms between the GTEx aging and the “ICM_vs_NF” and “DCM_vs_NF” were further visualized as a dot plot using the enrichplot R package. The genes that commonly contributed to the enrichment of the “Mitochondrial inner membrane” GO CC term for the human heart failure comparisons were visualized as heatmaps of scaled normalized expression counts. Similarly, for the GTEx aging GSEA results, the core enrichment genes of “Mitochondrial inner membrane” GO CC term was visualized as a heatmap of scaled Transcript Per Million (TPM) counts across aging. Genes were hierarchically clustered with a Euclidean distance metric using complete linkage.

#### *In situ* muscle force

For study 1, maximal forces were performed as described^58^ using an Aurora Scientific 305C servomotor (Aurora, Ontario, Canada) at baseline and after 7 weeks of treatment. Briefly, each mouse was anesthetized with isoflurane (4% for induction and ∼2% for maintenance) and laid on its side on a temperature-controlled platform maintained at 37°C. The right knee was clamped in place and the foot was secured to a footplate with the ankle positioned at 90°. The tibial nerve was stimulated with a Grass Instruments S88X stimulator (Astro-Med, Inc., West Warwick, Rhode Island, USA) at an optimal voltage (1.5 V) using percutaneous electrodes. Maximal tetanic torque was assessed by a force-frequency curve, where the muscle was stimulated every other minute at frequencies from 10 Hz to 200 Hz.

#### Echocardiography of aging studies

At baseline and after 8 weeks of treatment mice in study 1 were anesthetized with isoflurane (4% for induction and ∼2% for maintenance), and echocardiography was performed using The Vevo 3100 preclinical imaging system, Vevo Imaging Station, and MS400 probe from VisualSonics (Toronta, Canada). The mice were placed in a supine position and HR, respiration, core body temperature are monitored using Vevo Animal Monitoring system SM200 and Vevo Monitor App software from VisualSonics. Parasternal short axis view (PSAX) B-mode and M-Mode images were acquired. Echocardiography was analyzed using the Vevo LAB software. ECG and heart rate were monitored throughout the procedure with mouse heart rates being maintained in the range of 450–550 bpm at the LWL. To induce a high workload 3ug/g body weight dobutamine was injected to induce increased systolic function. High workload echocardiography performed once the heart rate increased ∼100bpm and remained stable. All protocols were approved by the University of Washington Institutional Animal care and use Committee. Systolic functions including LV mass, EF, FS are quantified at rest (LWL) and high workload (stabilized post IP injection). Diastolic function was accessed by tissue velocity from tissue doppler apical four chamber view (detailed description described previously^51^). In brief, an average of at least 3 individual cardiac cycles without a respiration are used to calculate peak early (E′) and peak late (A′) mitral valve anulus tissue velocity. The results were exported to Microsoft Excel and statistical analysis and graphing is performed using GraphPad Prism 9.0 software.

#### Euthanasia and tissue handling

Mice from study 1 were euthanized after 16 h overnight fasting by cervical dislocation after endpoint measurement. Heart, muscle, brain, kidney and liver were immediately removed and weighed. A 2mm section was removed from ventricles for histology and the remaining tissue were snap frozen in liquid N2 to store for further analysis.

#### Transmission Electron Microscopy

The tissues from mice in study 2 were slightly teased apart in 0.1 M Sorenson’s Phosphate buffer and cut into 1 mm^3^ pieces. Samples were immersion fixed in 2% glutaraldehyde with 0.1 M Sorenson’s buffer (pH 7.3) at 4°C.

Tissues were postfixed in 1% osmium tetroxide (1 h), dehydrated in graded acetone (25%; 50%; 75%; 2 × 100%; 30 min each, RT) before being impregnated with increasing concentrations of epoxy resin (TAAB medium resin) in acetone (25%, 50%, 75%, 3 × 100%, all for 1 h each, RT). The samples were then embedded in 100% fresh resin and left to polymerize at 60°C for a minimum of 24 h.

All resin blocks were trimmed using a razor blade to form a trapezoid block face. Sections were cut in a longitudinal or transverse orientation on an ultramicrotome^59^ using a diamond knife. Semithin sections (0.5 μm) were stained with toluidine blue and viewed on a light microscope to verify orientation of tissue. Ultrathin sections (70 nm) were then cut and picked up onto copper grids. Sections were stained with 1% uranyl acetate (30 min) and 3% lead citrate (7 min). All sections were examined using an HT7800 120kV TEM (Hitachi). Digital micrographs were captured using an EMSIS Xarosa CMOS Camera with Radius software.

#### Morphological and statistical analyses from transmission electron microscopy images

Mitochondrial shape descriptors and size measurements were obtained using ImageJ (version 1.52i, National Institutes of Health, Bethesda, MD, USA) by manually tracing mitochondria from TEM images. Surface area (mitochondrial size) is reported in squared micrometres; perimeter in micrometers; Values were imported into Microsoft Excel and Prism 9.0 software (GraphPad Software, San Diego, CA, USA) for data analysis. Statistical significance was evaluated based on 99% confidence interval (CI) of the median.

#### Immunoblotting

Protein levels of mitophagy and fusion/fission proteins were determined in rat right ventricles. Approximately 5 mg of crushed RV were homogenized in 300 μl of 1X RIPA buffer supplemented with protease and phosphatase inhibitor coktails (Thermofisher, 78430 and 78428). The homogenate was centrifuged at 15,000 g for 15 min at 4°C. Protein content was determined using the DC protein assay (Bio-Rad, 500-0112).

Proteins were diluted, mixed with Laemmli buffer (1610747, Biorad) containing 0.1M DTT and boiled at 95°C for 5 min. For each sample, twenty micrograms of proteins were loaded into 4–15% Mini-PROTEAN TGX Stain-Free Protein Gel stain-free (Biorad, 4568086), electrophoresed by SDS-PAGE, and then transferred to polyvinylidene fluoride membranes (1704156, Biorad). A stain-free blot image was taken using the ChemiDoc Touch Imaging System for total protein. Membranes were blocked in 5% BSA in Tris-Buffered Saline containing 0.1% Tween 20 (TBS-T) for 1h at room temperature. Membranes were incubated overnight with the following primary antibody diluted in blocking buffer: anti-phospho ubiquitin (Millipore, ABS1513-1, 1:1000), anti-OPTN (Proteintech, 10837-1-AP, 1:1000), anti-LAMP1 (Abcam, AB24170-10001), anti-DRP1 (Cell Signaling, 8570,1:1000) and anti-MFN1 (Proteintech, 13798-1-AP, 1:1000). After washing, membranes were incubated with HRP-conjugated secondary antibodies (Abcam Ab6728 or Ab6721, 1:10000) diluted in blocking buffer 1 h at room temperature. Signals were detected using enhanced chemiluminescence substrate (Biorad, Clarity ECL substrate, 170–5,060) using the ChemiDoc Touch Imaging System. All images were analyzed using the ImageLab software (Biorad). For each sample, the ECL signal for the protein of interest was normalized to the intensity of the stain-free blot image of the corresponding sample (i.e., the intensity of the stain-free blot image was used as loading control).

#### RNA extraction from mouse heart OCT samples

Excess OCT was trimmed away from the frozen tissues block with a sterile scalpel blade. The tissues were cut into sections of 20 μM using a cryostat previously cleaned with a RNAse away solution. 10 sections per sample were collected in microtubes containing 1mL of Trizol. The RNA was isolated using the TRIZOL/Chloroform extraction method.

Mouse heart aging RNA-seq data RNA was quality controlled with Agilent Fragment Analyzer System. Library preparation was performed by Strand-specific cDNA library, purification of poly-A containing mRNA molecules, mRNA fragmentation, random primed cDNA synthesis (strand-specific), adapter ligation and adapter specific PCR amplification. RNA-seq run was performed with the NovaSeq6000 using S4 flowcells with 2x150bp (>30 million read pairs (+/− 3%) per sample).

Data were grouped into four categories based on experimental conditions: Young with 4 samples, Young treated with UA (Young_UA) with 5 samples, Old with 6 samples, and Old treated with UA (Old_UA) with 3 samples. QC was performed as described for the human heart failure study above. High quality RNA-seq read pairs were mapped to the Ensembl mouse GRCm38 reference genome, using a transcriptome annotation GTF file (version GRCm38.94). An in-house RNA-sequencing alignment pipeline that utilizes the STAR aligner version 2.6.1c was applied. For the alignment process, a bespoke index was built using the annotation file, and an expected read length of 151 bps. Read counting was performed on the gene-level, using htseq-count. To inspect the alignment results, MultiQC version 1.8.dev0 was applied. Read counts were normalized using the variance stabilizing transformation method, implemented in DESeq2. Principal Component Analysis (PCA) was performed using the normalized read counts of the 1,000 most variable genes (mvgs) using base R (prcomp() PCA function), and visualized using the R package ggplot2. Differential expression analysis was applied between “Old” and “Young” conditions, using “Young” as reference dataset (referred as “Old vs. young”), and between “Old_UA” and “Old” conditions, using “Old” as reference dataset (referred as “Old UA vs. Old”). Before the DE analysis, a pre-filtering step was applied to remove low abundance mRNA measurements, by excluding genes with less than 10 total counts across samples. Additionally, an independent filtering procedure of DESeq2 was enabled, to filter out genes with very low counts that are unlikely to show significant alterations in gene expression. Gene symbols (MGI nomenclature), descriptions, and biotypes were matched to Ensembl GTF ids by using the R package biomaRt. v. 2.46. *p*-values were corrected for multiple-testing using the BH method.^57^

Gene Ontology Cellular Components (GO CCs) database was used for GSEA enrichment of the mouse heart aging and rat heart failure datasets. The enrichment analysis was performed for each DEA comparison separately, using the R package ClusterProfiler. For each comparison, the gene’s log2 fold change was multiplied by the respective *p*-value (log2(Fold Change) ∗ -log10(*p*-value)), and genes were ranked by the resulting value. Consequently, the enrichment of GO CCs gene sets among up- or-down regulated genes was statistically tested. The minimum and maximum gene set sizes were set to 10 and 500, respectively. The resulting *p*-values were adjusted using the BH method. Terms with an adjusted *p*-value ≤0.05 were considered as statistically significant and were further characterized as activated or suppressed based on the respective NES sign (activated for NES >0 and suppressed for NES <0). For the mouse heart aging dataset, activated GSEA GO CCs of the “Old UA vs. old” comparison were further filtered, keeping only the terms that were also significantly suppressed in the “Old vs. young” comparison.

#### Mitochondrial respiration assay

Isolated cardiac mitochondria were tested for respiration using Oxygraph 2k dual respirometer (Oroboros Instruments, Innsbruck, Austria). For the substrate-uncoupler-inhibitor titration (SUIT) protocol, 10 μM cytochrome *c* was added to each chamber to allow measurement of respiration in isolated mitochondria without limitation by membrane damage occurring during isolation. Approximately 100 μg heart mitochondrial homogenate was added to each 2 mL chamber. State three fatty acid oxidation was measured by addition PC (palmitoyl carnitine as substrate (Figure S3A). State three CI&CII respiration was stimulated by adding CI (Pyruvate, Malate, Glutamate) (Figure S3B) prior to stimulation with ADP followed by succinate for State 3 respiration (Figure S3C).

#### Echocardiography of heart failure study

To assess the cardiac remodeling and function following the MI, three echocardiography were performed 4 days, 1 month and 2 months after starting treatment. The first examinations were used to exclude rats with small infarcts and limited reduction of the EF.

Rats were anesthetized with 4% isoflurane in 50% oxygen-50% air mixture and maintained with 2–2.5% isoflurane during the procedure. The animals were placed in a supine position on a heating pad, thorax-shaved and imaged using a digital ultrasound system (Vivid 7,GE Medical Systems) equipped with a 10-Mhz phased-array and 13Mhz linear-array transducer. Standard B-mode (Brightness-mode) and M-mode (Motion-mode) images of the heart were obtained in the two-dimensional (2D) parasternal long axis view (PLAX). FS and EF were calculated using the LV dimensions parameters previously described and the following equations:

$$\text{FS}=\text{fractional}\;\text{shortening}=\left[\frac{LVIDd-LVIDs}{LVIDd}\right]x\;100\text{.}$$

$$\text{EF}=\text{ejection}\;\text{fraction}=\left(\frac{\left(end\;diastolic\;volume\;–\;end\;systolic\;volume\right)}{end\;diastolic\;volume}\right)X\;100$$

The isovolumic relaxation time, ratio of peak velocity of early (E) and late (A) waves of the mitral flow during diastole and cardiac output were calculated by Doppler-echocardiography only for the last examinations. All parameters were measured and averaged from three consecutive cardiac cycles under stable conditions by a single blinded trained operator.

#### Rat heart collection

One day after the last dosing, overnight-fasted rats were anesthetized with 2.5% isoflurane in 50% O_2_ 50% air mixture followed by an injection of buprenorphine (0.03 mg/kg). Hearts were stopped in diastole (KCL 2M, 1 mL/kg) by jugular injections. The atria were removed, the hearts were washed in cold PBS and quickly sponged to remove the liquid from the ventricles. LV was separated from the remaining part of the heart, weighed and incubated in NBF 10% for 48h. Hearts were transferred in ethanol 70%, cut in three transverse sections and embedded in paraffin in three different blocks, deriving from apical, middle and basal LV regions (Group 1, *N* = 10, group2 and *N* = 20).

#### Infarct area and fibrosis quantification

Briefly, tissues block from the apical, middle and basal LV regions were cut into sections of 5 μM using a microtomes. Sections were stained with Sirus-RED (SR). To evaluate the extent of interstitial fibrosis, the SR area was quantified with the digital image analysis Visiopharm software. The infarct area was measured using ImageJ and averaged per animal.

#### Rat heart RNA extraction

The protocol was performed according to the manufacture instructions using the kit RNeasy FFPE (Qiagen, 73504). Tissues blocks were cut into sections of 20 μM and collected in microtubes. Samples were incubated for 3 min at 56°C in a deparaffinization solution (Qiagen, 19093). A step of digestion with Proteinase K was performed followed by a treatment with DNAses. RNA was purified using the RNeasy MinElute column and eluted in 30uL of RNAse free water.

#### Rat heart RNA-seq

Data were grouped into three categories based on experimental conditions: Vehicle (Sham) with 4 samples, MI with 4 samples, and MI + UA with 5 samples. QC was performed as in the mouse RNA-seq study. High quality RNA-seq read pairs were mapped to the Ensembl rat mRatBN7.2 reference genome using a transcriptome annotation GTF file (version mRatBN7.2.108). PCA was performed as in the muse RNA-seq study and highlighted a clear outlier “MI_UA” condition) which was excluded from downstream analysis (visualization not shown). Differential expression analysis was applied between “MI” and “Sham” conditions, using “Sham” as reference dataset (referred as “MI vs. sham”), and between “MI + UA” and “MI” conditions, using “MI” as reference dataset (referred as “MI UA vs. MI”). DEA and GSEA were performed as described in the human RNA-seq section (see above). RNA-seq was performed as in the mouse RNA-seq study, with the exception of applying rRNA depletion instead of poly-A containing mRNA purification.

#### Heart immunohistochemistry

Tissue blocks from the middle LV regions were cut into sections of 4 μM using a microtome. Sections were deparaffinized in xylene, rehydrated in descending alcohol baths and brought into distilled water. Permeabilization was performed by 15 min incubation in 0.1% Triton X-100 at 37°C. Slides were washed with PBS, containing 0.1% Triton X-100 and blocked 1 h in 5% goat serum (Jackson Immuno Research, 005-000-121) 3% BSA (Pan Biotech, P06-1391100). Rabbit anti-phospho-ubiquitin, (Millipore, ABS1513-and mouse anti-VDAC (Abcam, ab14734) were diluted to 1/50 and 1/500 respectively before incubation at 4°C overnight. Sections were washed in TBST and incubated for 2h with the secondary antibodies: Alexa 555 goat anti-rabbit (Invitrogen, A21428) and Alexa 488 goat anti-mouse (Invitrogen, A11001). Slides were mounted with Vectashield Antifade Mounting Medium (Adipogen, H-1500). Images were acquired using a confocal microscope (Zeiss, LSM-880).

Images were analyzed using ImageJ. Background was removed by applying an intensity threshold. After converting the images in mask, particles were quantified after filtering to remove non-specific signal determined from negative controls.

#### Plasma sphingolipid analysis

For absolute quantification of sphingolipids (Sph), plasma samples (25 μL) were extracted by the addition of 200 μL of ice-cold MeOH containing the internal standards (*n* = 9). This solution was vortexed 600 s and then centrifuged for 15 min at 4°C and 4500g. The resulting supernatant was transferred to a 96-well plate and injected into the LC-MS/MS system. Ten-point calibration curves were generated following the same procedure as for the samples: by addition of 200 μL of MeOH containing the IS mixture to each pre-prepared calibrator (25 μL), containing the increasing amount of each standard.

Extracted samples were analyzed by Liquid Chromatography coupled to tandem mass spectrometry (LC - MS/MS) in positive ionization mode using a TSQ Altis triple quadrupole system (QqQ) interfaced with a Vanquish UHPLC system (Thermo Fisher Scientific). Chromatographic separation was carried out in a Zorbax Eclipse plus C18 column (1.8 μm, 100 mm × 2.1 mm I.D) (Agilent technologies). Mobile phase was composed of A = 5 mM ammonium formate and 0.2% formic acid in water and B = 5 mM ammonium formate and 0.2% formic acid in MeOH at a flow rate of 600 μL/min. Column temperature was 40°C and sample injection volume 4μL. The linear gradient elution starting from 80% to 100% of B (in 8 min) was applied and held until 14 min. The column was then equilibrated to initial conditions. Optimized HESI source parameters were set as follows: voltage 3500 V in positive mode, Sheath Gas (Arb) = 60, Aux Gas (Arb) = 15, Sweep Gas (Arb) = 1 and Ion Transfer Tube Temperature 380°C. Nitrogen was used as the nebulizer and Argon as collision gas (1.5 mTor). Vaporizer Temperature was set to 350°C. Optimized compound-dependent parameters were used for data acquisition in timed- Selected Reaction Monitoring (t-SRM) mode.

Raw LC-MS/MS data acquired in t-SRM mode was processed Clinical Trace Finder software (version 4.1, Thermo Fisher Scientific). Quantification of sphingolipids was based on EIC (Extracted Ion Chromatogram) areas for the monitored SRM transitions, corresponding calibration curves and the corresponding (or structural analogues) stable isotope-labeled internal standard (IS) response. Linearity of the standard curves was evaluated for each metabolite using ten calibration points; in addition, peak area integrations were manually curated and corrected when necessary.

### Quantification and statistical analysis

Unless otherwise indicated, statistical analyses were performed GraphPad Prism 9.0 software. Data are reported as mean ± SEM. Statistically significant differences between 2 independent groups were determined with Student’s unpaired t-test. Unpaired t-test with Welch’s correction were used to determine significance for pairs (*p* < 0.05). Statistically significant differences between three independent groups were determined by one-way ANOVA test followed by Tukey’s multiple comparison test. For group analysis involving two variables, a two-way ANOVA followed by Tukey’s multiple comparison test was performed. *p* values less than 0.05 were considered significant. Statistical analysis of RNA-seq experiments is described in the corresponding sections. Sample sizes are indicated in Figure Legends.

For human ceramide data analysis, ceramide data completeness was assessed by summarizing the visits for each subject, ensuring that only subjects with complete visit data were included in the subsequent analysis. Subjects with incomplete data across visits were identified and excluded to maintain the integrity of the statistical analysis, and concentrations were scaled to standardize the data. The statistical analysis was conducted to evaluate the effect of the treatment on the changes in ceramide concentrations over time, adjusted for baseline measurements. A mixed-effects model was employed to account for the repeated measures within subjects across visits. The model included fixed effects for treatment, visit, and their interaction, and a random effect for the subject to model the within-subject correlations. The model was specified as follows:

Concentration∼Concentration_baseline+treatment+(1|Subject)

ANCOVA (Analysis of Covariance) was utilized to determine the adjusted means of ceramide concentrations, comparing the treatment group against the placebo across visits. The response variable in the model was the concentration of each ceramide, with the baseline concentration as a covariate, and treatment group as the main factor.

Contrasts were set up to test differences between changes from baseline to each subsequent visit within and between treatment groups. Adjustments for multiple comparisons were made using the Benjamini-Hochberg method to control the false discovery rate. Significant differences were identified based on adjusted *p*-values. Post-hoc tests were conducted using estimated marginal means to compare the treatment effects at each time point directly. Statistical analysis was conducted using R. The analysis was repeated using C24:0 as a normalizer (ratios of concentrations).

Whenever possible, individual values are displayed in graphs. When individual values could not be displayed, sample sizes are indicated in Figure Legends.

## Supplemental information

Document S1. Figures S1–S5

Table S1. Gene ontology cellular component categories in human datasets from Figure 1

Table S2. Raw echocardiography results from the rat heart failure study in Figure 2

Table S3. GO cellular component categories in the rat heart failure study from Figure 3

Table S4. GO cellular component categories in the mouse cardiac aging study from Figure 5

Table S5. List of sphingolipid species measured in the plasma of mice as in Figure 6

Table S6. List of sphingolipids in human plasma as in Figure 6
