# Supplementary material for: Urolithin A provides cardioprotection and mitochondrial quality enhancement preclinically and improves human cardiovascular health biomarkers
Source: iScience. 2025 Jan 14;28(2):111814. doi: 10.1016/j.isci.2025.111814 (PMC11875685; doi:10.1016/j.isci.2025.111814)
Supplement: Document S1. Figures S1–S5 [file mmc1.pdf]

iScience, Volume *28*

Supplemental information

Urolithin A provides cardioprotection

and mitochondrial quality enhancement preclinically

and improves human cardiovascular health biomarkers

Sophia Liu, Julie Faitg, Charlotte Tissot, Dimitris Konstantopoulos, Ross Laws, Guillaume Bourdier, Pénélope A. Andreux, Tracey Davey, Hector Gallart-Ayala, Julijana Ivanisevic, Anurag Singh, Chris Rinsch, David J. Marcinek, and Davide D'Amico

Supplementary Material

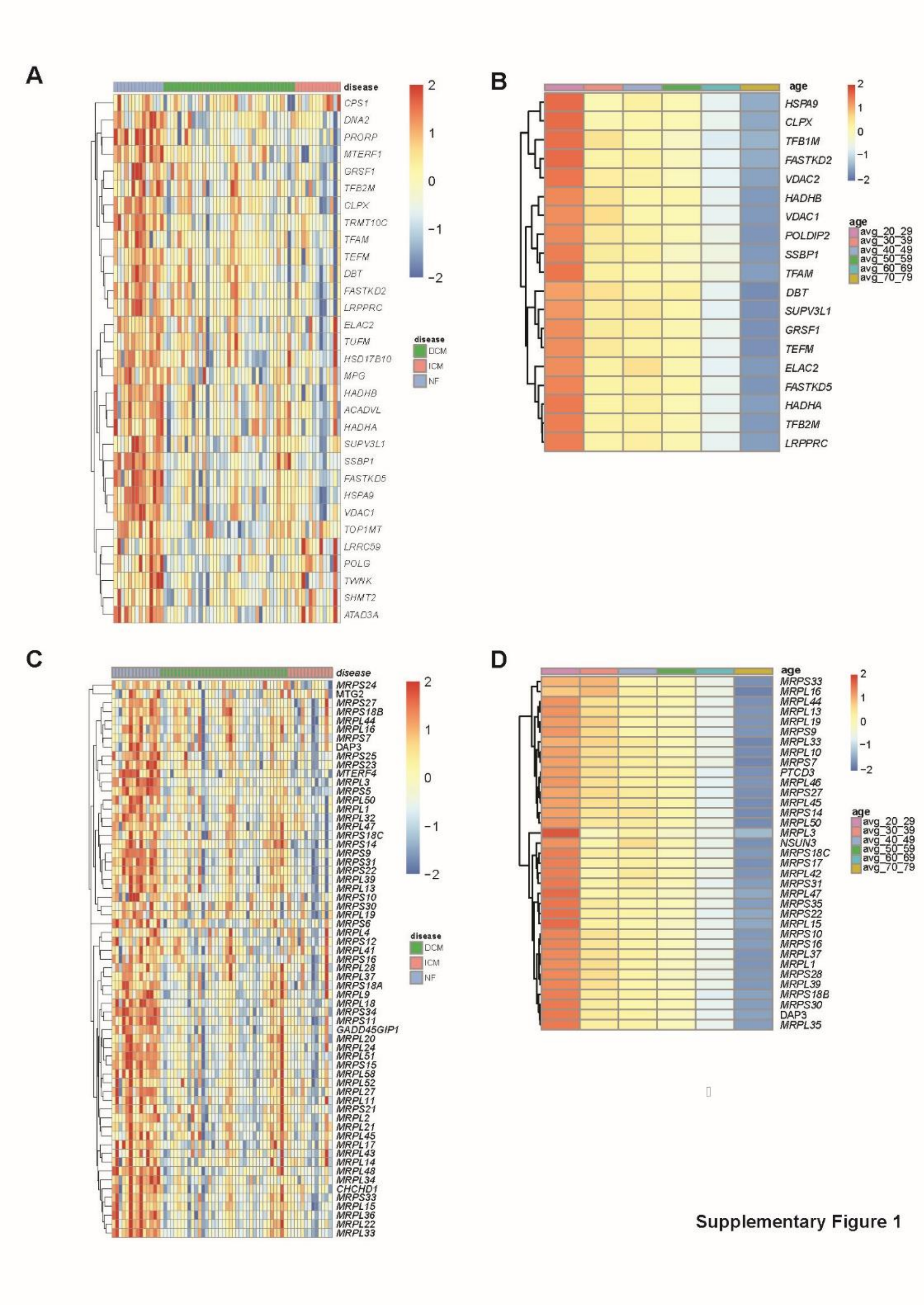

Supplementary Figure 1: **Mitochondrial genes commonly dysregulated in human heart aging and disease, related to Figure 1.**

A) Heatmaps of scaled normalized expression counts (z-scores) of genes that contributed to the enrichment of the "Nucleoid" GO CC term in human DCM and ICM compared to Non-Failing, as in Fig.1C. Rows represent genes hierarchically clustered using a Euclidean distance metric and complete linkage. Columns represent individual samples from each condition.

B) Heatmaps of the scaled Transcripts Per Million (TPM, as z-scores) of core enrichment genes contributing to the " Nucleoid " GO CC term in the human cardiac aging study (GTEx Aging), as in Fig. 1D.

C) Heatmaps of scaled normalized expression counts (z-scores) of genes that contributed to the enrichment of the "Organellar ribosomes" GO CC term in human DCM and ICM compared to Non-Failing, as in Fig.1C. Rows represent genes hierarchically clustered using a Euclidean distance metric and complete linkage. Columns represent individual samples from each condition.

D) Heatmaps of the scaled Transcripts Per Million (TPM, as z-scores) of core enrichment genes contributing to the " Organellar ribosomes" GO CC term in the human cardiac aging study (GTEx)

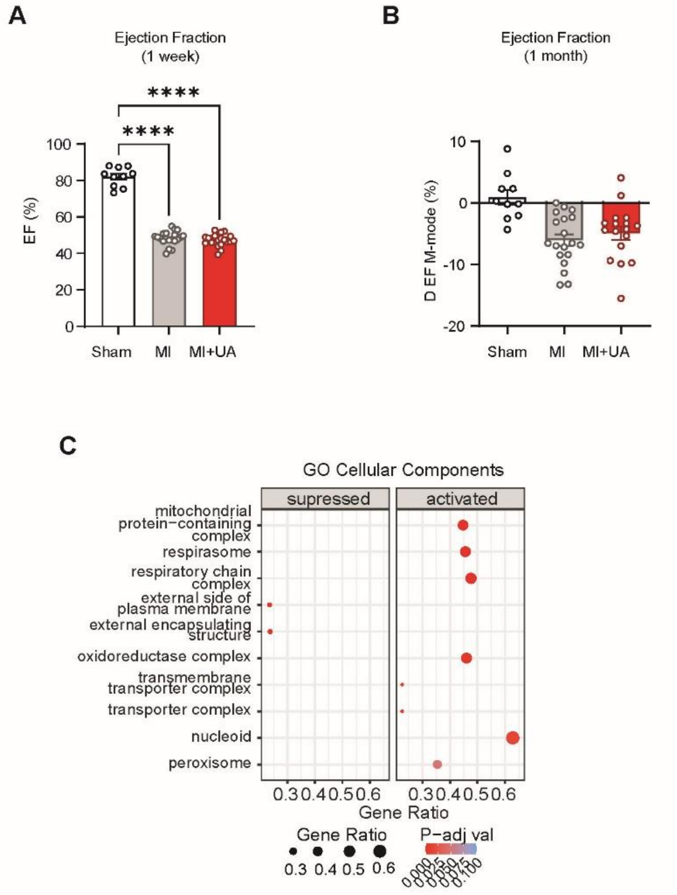

Supplementary Figure 2

Supplementary Figure 2 : **Urolithin A improved cardiac function and mitochondrial health in model of HFrEF, related to Figure 2 and 3**

A) Ejection fraction measured at the 1-week time point indicating a significant reduction in systolic function following surgery, compared to sham controls. ****p < 0.001 after one-way ANOVA. Error bars represent mean ± SEM.

B) Ejection fraction measure as in Fig. 4C at the 1-month time point.

C) Dot plot summarizing the 8 GO CCs highlighted in (A, Upper panel) and (B). The GO CCs are sorted by the statistical significance of their activation in the "MI UA vs MI" comparison (represented as a Benjamini-Hochberg-adjusted p-value, or P-adjusted value). The size of each dot corresponds to the Gene Ratio, while the color indicates the statistical significance of the

enrichment (represented as a Benjamini-Hochberg-adjusted p-value, or P-adjusted value). The "Gene Ratio" refers to the ratio of "core enrichment" genes within a specific gene set relative to the total number of genes in that set, providing a measure of the enrichment's extent. "Core enrichment" genes contribute to the enrichment signal in a gene set.

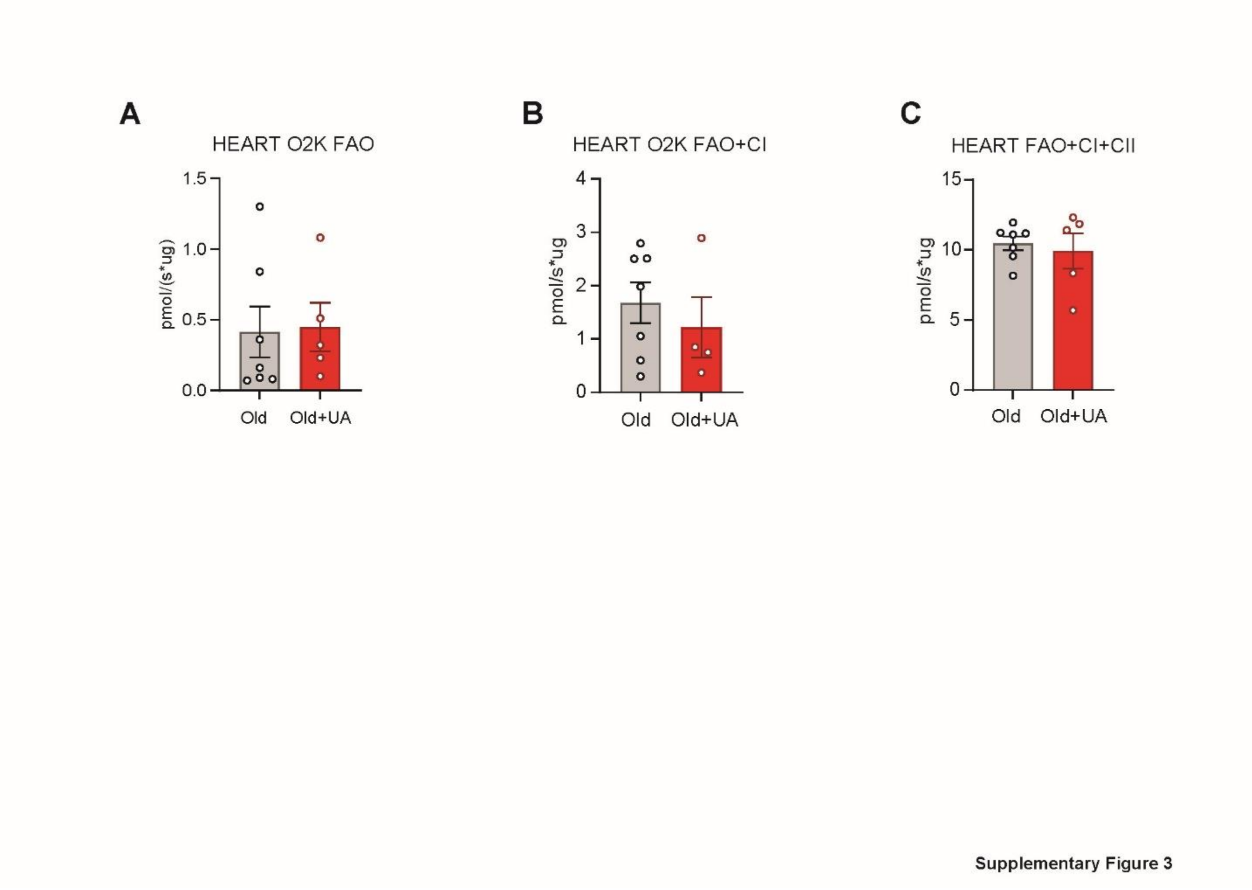

***Supplementary Figure 3*. Mitochondrial respiration in old mice treated with Urolithin A, related to Figure 4.**

A) State three fatty acid oxidation measured by addition PC (palmitoyl carnitine as substrate) in isolated heart mitochondria from the cardiac aging study in Fig. 3.

B-C) State three CI&CII respiration was stimulated by adding CI (Pyruvate, Malate, Glutamate) prior to stimulation with ADP (B) followed by succinate for State 3 respiration (C).

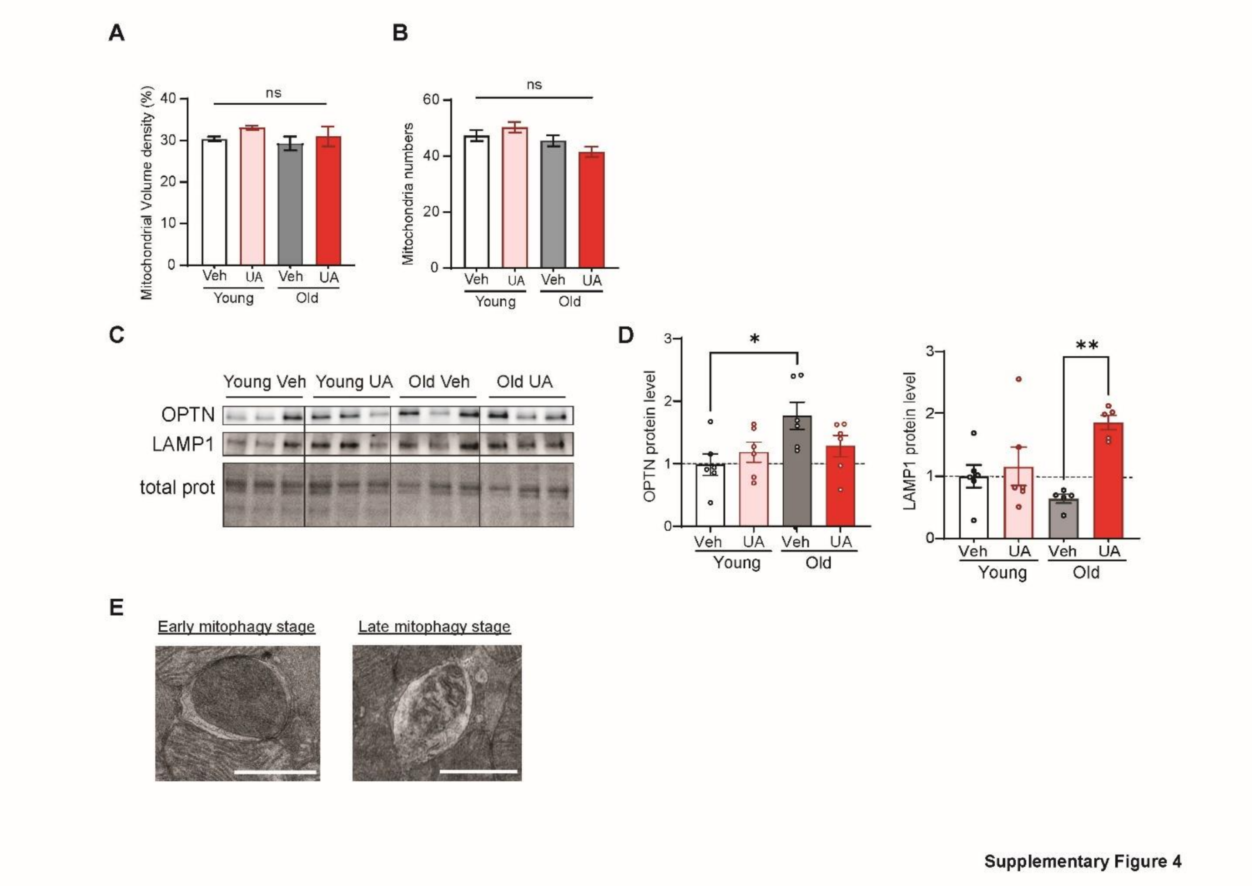

***Supplementary Figure 4*. Mitochondrial phenotyping of young and old mice after UA administration related to Figure 5**

A) Mitochondrial volume density quantification of transversal images from young and old mice administered with either vehicle or UA for 2 months, expressed as percentage over total area in the indicated animals.

B) Mitochondrial numbers quantify in each individual images per animal per group (N=3 animals/group). p value. $^*p<0.05$; *** $p<0.005$; $^{****}p < 0.001$ after one-way ANOVA. Error bars represent mean ± SEM.

C-D) Representatives immunoblot of optineurin (OPTN), LAMP1 and stain free blot of total proteins (C) and quantification of OPTN (D) and LAMP1 (F) normalized over their corresponding total protein stain free blot (N=6 animal/group). p value. $^*p<0.05$; ** $p<0.01$; after one-way ANOVA. Error bars represent mean ± SEM.

E) Representative images of early and late stage mitolysosome use to perform the mitophagy event quantification described in Fig.5I.

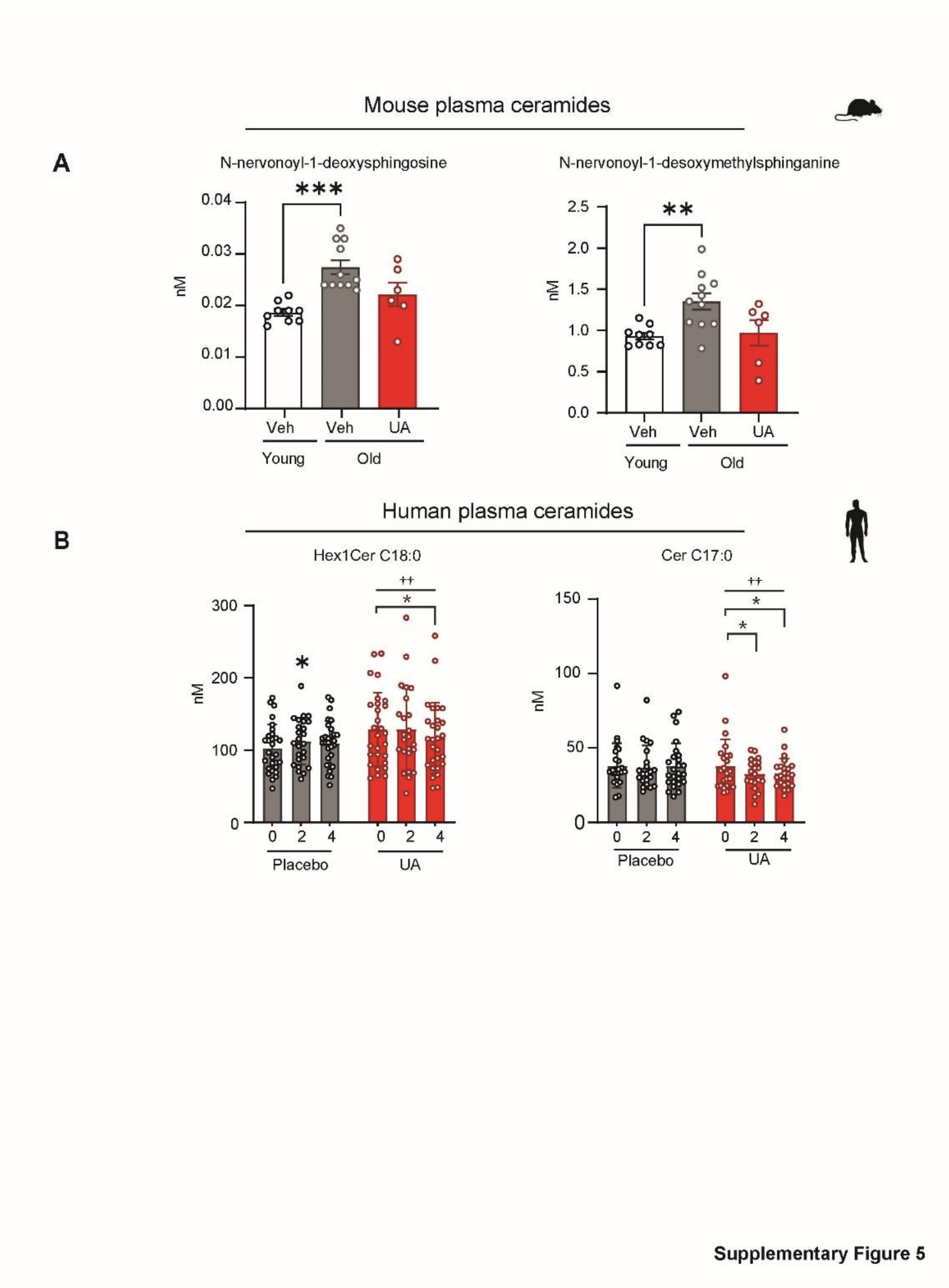

Supplementary Figure 5. **Urolithin A treatment reduces ceramides species in mice and humans, related to Figure 6.**

A) Concentrations of N-nervonoyl-1-deoxysphingosine and N-nervonoyl-1-desoxymethylsphinganine in plasma of the indicated mice groups. (N=5-11) p value. ** $p<0.01$; *** $p<0.005$ after one-way ANOVA. Error bars represent mean ± SEM.

B) Concentrations of ceramides Hex1Cer C16:0 C18:0 and C24:1 and ceramides Cer17:0 in plasma of healthy elderly subjects supplemented with either Placebo or Urolithin A (1g) at baseline (0), after 2 months or 4 months. *$p<0.05$; ** $p<0.01$; after ANCOVA. Error bars represent mean ± SEM.
Cer: ceramides; Hex1Cer: Hexosyl-1-ceramides.

***Supplementary Table 1:* Gene Ontology Cellular Component categories in human datasets from Figure 1.**

List of significant Gene Ontology Cellular Component categories in human datasets from Figure 1. Tabs indicate all significant gene set in each dataset (FULL) and the common significant gene sets between datasets. NES = Normalized Enrichment Score. "Gene Ratio": ratio of "core enrichment" genes within a specific gene set relative to the total number of genes in that set.

***Supplementary Table 2:* Raw echocardiography results from the rat heart failure study in Figure 2.**

Raw echocardiography results from the rat heart failure study in Figure 2. Table indicated the % change in FS, EF and IVRT in individual rats from each experimental condition.

***Supplementary Table 3:* GO Cellular Component categories in the rat heart failure study from Figure 3.**

List of significant Gene Ontology Cellular Component categories in the rat heart failure study from Figure 4, indicating all significant gene sets that are both induced comparing UA-treated and UA untreated heart failure rats (MI UA versus MI) and suppressed in heart failure rats versus controls (MI versus Sham). NES = Normalized Enrichment Score. "Gene Ratio": ratio of

"core enrichment" genes within a specific gene set relative to the total number of genes in that set.

Supplementary Table 4: **GO Cellular Component categories in the mouse cardiac aging study from Figure 5.**

List of significant Gene Ontology Cellular Component categories in the mouse cardiac aging study from Figure 2, indicating all significant gene sets that are both induced comparing UA-treated old animals versus Old untreated animals and suppressed in old versus young mice. Top categories are represented in Fig. 2D. NES = Normalized Enrichment Score. "Gene Ratio": ratio of "core enrichment" genes within a specific gene set relative to the total number of genes in that set.

Supplementary Table 5: **List of sphingolipid species measured in the plasma of mice as in Fig.6.**

List of sphingolipid species measured in the plasma of mice as in Fig.5. Table indicates the statistical significance of each species (one way ANOVA) and fold changes, in old mice compared to young mice and old mice treated with UA compared to old mice.

Supplementary Table 6**: List of sphingolipids in human plasma as in Fig.6.**

List of sphingolipid species measured in the plasma of human plasma from as in Fig.5. Table indicates the statistical significance of each species (ANCOVA) and fold changes. Comparison applied include changes at 2 months (V2) over baseline (V1); changes at 4 months (V3) over baseline); and time course effects (V1 to V2 to V3).
